# Feasibility cluster randomised controlled trial of a within-consultation intervention to reduce antibiotic prescribing for children presenting to primary care with acute respiratory tract infection and cough

**DOI:** 10.1136/bmjopen-2016-014506

**Published:** 2017-05-09

**Authors:** Peter S Blair, Sophie Turnbull, Jenny Ingram, Niamh Redmond, Patricia Jane Lucas, Christie Cabral, Sandra Hollinghurst, Padraig Dixon, Tim J Peters, Jeremy Horwood, Paul Little, Nick A Francis, Anna Gilbertson, Catherine Jameson, Alastair D Hay

**Affiliations:** 1 Centre for Child and Adolescent Health, School of Social & Community Medicine, University of Bristol, Bristol, UK; 2 Centre for Academic Primary Care, School of Social and Community Medicine, University of Bristol, Bristol, UK; 3 National Institute for Health Research Collaborations for Leadership in Applied Health Research and Care West (NIHR CLAHRC West), University Hospitals Bristol NHS Foundation Trust, UK; 4 School for Policy Studies, University of Bristol, Bristol, UK; 5 School of Social and Community Medicine, University of Bristol, Bristol, UK; 6 School of Clinical Sciences, University of Bristol, Bristol, UK; 7 Department of Primary Care & Population Sciences, University of Southampton, Southampton, UK; 8 Division of Population Medicine, Cardiff University, UK

**Keywords:** Randomised controlled trial (RCT), Feasibility, Children, Respiratory tract infections (RTIs), Primary care, Cough, Antibiotics (or Anti-microbial agents)

## Abstract

**Objective:**

To investigate recruitment and retention, data collection methods and the acceptability of a ‘within-consultation’ complex intervention designed to reduce antibiotic prescribing.

**Design:**

Primary care feasibility cluster randomised controlled trial.

**Setting:**

32 general practices in South West England recruiting children from October 2014 to April 2015.

**Participants:**

Children (aged 3 months to <12 years) with acute cough and respiratory tract infection (RTI).

**Intervention:**

A web-based clinician-focussed clinical rule to predict risk of future hospitalisation and a printed leaflet with individualised child health information for carers, safety-netting advice and a treatment decision record.

**Controls:**

Usual practice, with clinicians recording data on symptoms, signs and treatment decisions.

**Results:**

Of 542 children invited, 501 (92.4%) consented to participate, a month ahead of schedule. Antibiotic prescribing data were collected for all children, follow-up data for 495 (98.8%) and the National Health Service resource use data for 494 (98.6%). The overall antibiotic prescribing rates for children’s RTIs were 25% and 15.8% (p=0.018) in intervention and control groups, respectively. We found evidence of postrandomisation differential recruitment: the number of children recruited to the intervention arm was higher (292 vs 209); over half were recruited by prescribing nurses compared with less than a third in the control arm; children in the intervention arm were younger (median age 2 vs 3 years controls, p=0.03) and appeared to be more unwell than those in the control arm with higher respiratory rates (p<0.0001), wheeze prevalence (p=0.007) and global illness severity scores assessed by carers (p=0.045) and clinicians (p=0.01). Interviews with clinicians confirmed preferential recruitment of less unwell children to the trial, more so in the control arm.

**Conclusion:**

Differential recruitment may explain the paradoxical antibiotic prescribing rates. Future cluster level studies should consider designs which remove the need for individual consent postrandomisation and embed the intervention within electronic primary care records.

**Trial registration number:**

ISRCTN 23547970

**UKCRN study ID:**

16891

**Strengths and limitations of this study**

- Recruitment was successful with robust data collection and few missing values.
- We adequately resourced the trial, achieving excellent follow-up with little attrition.
- We reduced variation in practice recruitment levels, compared with our previous cohort study.
- None of the intervention web sessions were abandoned and clinicians thought it was quick and easy to use compared with other interventions.
- Clinicians valued the personalised leaflet as an alternative to a prescription, although carers were less enthusiastic.
- The intervention was designed to be used as an antibiotic treatment decision aid, but it became apparent that some clinicians only used the intervention after deciding treatment.
- Using the intervention added around 5 min to consultation time. Some of this time was to record research data and consideration needs to be given as to whether these data can be collected outside the consultation.
- The intervention was a stand-alone system and might be more acceptable and easier to use if embedded within the existing practice records system.
- Differential recruitment was quite marked, in particular the intervention children were significantly more unwell. A future trial design needs to minimise or avoid this postrandomisation recruitment bias.
- The control clinicians used the same web-based data collection system during consultation to record study data. Thus, our data collection in the controls may have inadvertently increased risk perception as well as providing the ‘test’ conditions to encourage a Hawthorne effect.

## Introduction

Respiratory tract infections (RTIs) in children are extremely common and costly to service providers, families and employers in the UK.^1 2^ Clinicians often prescribe antibiotics ‘just in case’, if they feel uncertain about patient health or legal outcomes.^3^ Clinical uncertainty in primary care regarding the diagnosis and best management of RTIs has led to the inappropriate use of existing antibiotics, which, combined with the slowing in development of new antibiotics, is associated with antimicrobial resistance.^4–6^ This has been described as one of the greatest challenges to public health today,^7 8^ with the World Economic Forum placing antibiotic-resistant bacteria on the 2014 Global Risks List.^9^ The 5-year National Institute for Health Research (NIHR)-funded ‘TARGET’ Programme for Applied Research was set up in 2010 to derive new knowledge to improve the management of antibiotics given to children presenting to primary care with RTIs and cough.

Systematic reviews from this programme^10 11^ found that passive strategies targeting only parents, such as waiting room posters or pamphlets, do not appear to alter prescribing rates significantly. The most effective interventions involved targeting both parents and clinicians during a consultation, using automatic computer prompts for evidence-based prescribing, involving clinicians in the design of the intervention, using printed materials with actionable information and integrating interventions into routine clinical processes. Qualitative research found that communication within consultations often failed to meet parents’ needs: information on symptom relief was lacking, safety-netting advice was too vague to be useful and parents’ concerns went unaddressed.^3 12–15^ Clinicians revealed that prognostic uncertainty is an important driver of antibiotic prescribing, and would like information to help them identify the children at risk of future illness deterioration.^3 16 17^

Using a large multicentre, prospective cohort study (over 8300 children), we derived and internally validated a prognostic rule, using symptoms, signs and demographic characteristics to predict hospitalisation in the following 30 days among children presenting to primary care with acute cough and RTI.^18 19^ Findings from across the TARGET programme were synthesised to develop a complex intervention, designed to reduce antibiotic prescribing. The Children’s Cough (CHICO) feasibility trial reported here is the final element of the TARGET programme, the aim of which was to assess the recruitment and acceptability of the complex intervention and to understand whether it is feasible to conduct a larger trial.

## Methods

### Design

The CHICO feasibility trial was a primary care cluster randomised controlled trial (RCT) comparing a web-based intervention with usual care for children presenting to general practices with RTI and acute cough. The trial was approved by the North West-Haydock Research Ethics Committee, UK (reference number: 14/NW/1034, Trial registration: ISRCTN23547970, UKCRN study ID: 16891). The trial protocol^20^ was devised according to the SPIRIT guidelines^21^ for RCTs, reporting of the findings followed consolidated standards of reporting trials (CONSORT) guidelines for clustered trials^22^ and below is a brief summary of the methodology.

### Practise and patient recruitment

General practitioners (GPs) were invited to participate in the trial from locations in Bristol and the surrounding areas; the only exclusion criterion was for those few practicses using very outdated internet browsers. GPs and prescribing nurses (hereafter termed ‘clinicians’) were eligible to recruit children to the trial and informed consent was obtained from the parent or legal guardian (carers). Children were included if they were aged between 3 months and under 12 years and presenting with an RTI with acute cough of no more than 28 days duration prior to consultation. They were eligible if they presented with illnesses such as asthma (including those with infective exacerbations), epilepsy or diabetes. Children who required same day hospital assessment or admission were also included. Children were excluded if they were considered to have a high risk of serious infection (immune-compromised, cystic fibrosis, splenectomy).

### Randomisation and sample size

The allocation process was a one-off randomisation stratified for both practice size and prevalence of antibiotic prescribing using a proxy measure of amoxicillin suspension prescriptions (the main antibiotic used for children with cough) within the previous 12 months. As this was a feasibility trial, a formal (effectiveness-based) sample size calculation was not required, although we were interested in the recruitment rate to inform a larger trial design. Recruitment rates varied widely between practices in our previous cohort study,^19^ so we monitored recruitment levels closely and capped recruitment at no more than 30 patients per practice. Estimates from the cohort study suggested 15 practices in each arm of the trial would yield a pragmatic sample between 300 and 500 patients over a 7-month period.

### Intervention

Intervention development will be described in more detail elsewhere, although the paper reporting algorithm development has been published.^19^ In brief, findings from the TARGET programme were synthesised using the Precede-Proceed model of Green and Krueter^23^ as a framework. The active elements of the intervention were determined to be elicitation of parents’ concerns, active no-antibiotic messages to the clinician, reduction of clinician uncertainty and support for a no-antibiotic treatment response. These elements were provided using a within-consultation interactive web-based tool, which also provided a data collection tool. This tool delivered the following intervention elements:

1. Recording children’s symptoms and signs.
2. Elicitation and recording carers’ concerns.
3. For each consultation, children were identified as being at very low, normal or high risk of future hospitalisation; current National Institute for Health and Care Excellence guidelines on antibiotic associated with each risk strata were also provided.
4. A personalised printout was produced for carers of each child. Clinical observations and carers’ concerns recorded in the system were used to produce this personalised information leaflet that explained the best home-care strategies and reinforced important safety netting advice. The aim of this leaflet was to give clinicians a tangible treatment action other than (or in addition to) prescribing, to improve safety-netting information and to provide home-care support for carers.

### Data collection

Both control and intervention clinicians used the same study website to record carers’ consent, enter baseline and symptom data during the consultation and record their treatment decisions. Additionally, intervention clinicians were provided with background information about how the risk algorithm was developed, recorded carers’ concerns, were given on-screen the future hospitalisation risk of the child and provided carers with a personalised printout. Use of the intervention was assessed by recording the number of times the clinicians used the web-based intervention and time spent on each page of the website. Follow-up data were collected each week from carers until the child’s cough had resolved, or up to 8 weeks if not resolved. A medical record notes reviews were conducted at the recruiting practices to collect data relating to the 30 days following the recruitment consultation, including recruiting consultation timings, RTI-related antibiotic prescriptions, reconsultations and RTI-related hospitalisations.

### Qualitative interviews

Clinicians from both arms and carers from the intervention arm only were invited to participate in semi-structured interviews to explore their views of web-based data collection and the intervention. Interviews with carers were conducted either in the week following recruitment (to facilitate recall of the consultation) or after their child had recovered, to reflect on the whole follow-up data collection process. Purposive sampling was used to maximise variation in the sample of carers, including those with a range of child ages, home neighbourhood socioeconomic deprivation, illness severity scores and treatment outcomes. Clinicians were purposefully sampled to include variation in recruiting and prescribing levels.

### Health economics

The purpose of the economic analysis was to inform the feasibility and design of a within-trial economic evaluation alongside a larger RCT, including assessing web-based data collection as a means of gathering comprehensive resource use and quality of life data from carers. We adopted a health system (ie, National Health Service (NHS)) perspective for the analysis of the costs associated with resource use. We measured resources used from the time of recruitment until the earliest of either resolution or the end of the eighth week of follow-up. Health system resources used included GP consultations, use of out-of-hours services, NHS 111, walk-in centres and hospital and ambulance use. We examined quality of life using the Child Health Utility 9D (CHU-9D) instrument, a generic measure of paediatric quality of life validated for children aged 5–11 years. CHU-9D was included in the web-based tool for those children within the valid age bracket.

Carers were given the option of providing data using a paper version rather than online if preferred. Participants were sent text and email reminders followed by telephone calls if the data were not returned promptly.^24 25^ The length of the initial consultation was recorded electronically by the web-based tool and GP practice systems.

### Data analysis

Descriptive statistics were used to illustrate the characteristics of the carers, children and recruiting clinicians. For between-arm comparisons, categorical data were tested using χ^2^ or Fisher’s exact test if an expected cell value was <5, continuous data were plotted and, if not normally distributed, non-parametric tests (Mann-Whitney U test) were used. The use of baseline statistical tests to compare the arms is legitimate in the context of stratified cluster randomisation with subsequent (unconcealed) recruitment of individual participants. Such tests were used to ascertain if there was any evidence of differential recruitment of individuals across the trial arms beyond what would be expected by chance.

Qualitative interviews were audio-recorded, transcribed, anonymised and imported into NVIVO 10. Analysis of qualitative data began shortly after data collection started and was ongoing and iterative. An inductive thematic analysis approach was used to identify patterns and themes.^26^ An initial coding frame was developed and refined as new data were produced. Two researchers (CC and JH) double coded a subset of transcripts to inform the coding framework and ensure robust analysis.^27^

For both cost and quality of life data, the extent and nature of missing data was examined and compared between intervention and control practices. Descriptive statistics were used to explore resource use and cost by category and by arm to identify important cost drivers.

## Results

### Ascertainment and data collection

A total of 32 practices were recruited to the trial, 16 randomised to each arm. Within the practices, 104 clinicians signed up as recruiters and 64 recruited at least one child (18 nurses and 46 GPs). The target of 500 recruited children was achieved 1 month ahead of schedule (October 2014 to March 2015 inclusive); 542 children were invited to enter the trial and 501 agreed. Of the 41 not included, 18 (3.3%) declined, 19 (3.5%) did not meet the eligibility criteria and 2 (0.4%) were subsequently withdrawn by the carers. We obtained complete carer-reported follow-up data for 495 (98.8%) from the 501 children retained in the trial. Only two practices recruited no children; the median number of children recruited at each practices was 16 (range 0–30, IQR 4–29). Primary care medical record data were collected for 100% of the children. Web-based tool information was available for all but one of the 501 children.

### Sample description

The median age of the 501 children in the trial was 2 years (IQR: 1–4 years, full range: 3 months to 11 years), 49% were boys and there was no evidence of a difference in ethnicity when compared with the 2011 UK Census data nor with the prevalence of smoking in the household when compared with a 2013 Opinions and Lifestyle survey. However, using the postcode of the family residence, the median Index of Multiple Deprivation score in the trial (12.1) was markedly lower than national data (2010 Government statistics), suggesting children in this cohort were less deprived than children in the general population.

### Comparison between study arms

#### Recruitment

Although there were fewer clinicians in the intervention arm (46 vs 58 controls), they recruited 40% more children (292 vs 209 children). Table 1 shows a lower proportion of control group clinicians recruited children (p=0.02). In the intervention arm, a higher proportion of clinicians were nurses (28% vs 19% controls), who seemed to recruit at a higher rate. More than half the children in the intervention arm were recruited by nurses compared with less than a third among the controls, although both GPs (median 3 children vs 1 among controls, p=0.13) and nurses (median 12 children vs 3 among controls, p=0.08) in the intervention arm recruited at higher rates compared with the controls.

**Table 1:** Comparison of the clinician variables in the two arms of the trial

	Control		Intervention		
	n/N or median	% or (IQR)	n/N or median	% or (IQR)	p Value
Clinician profile					
Number of GCP trained clinicians	34/58	58.6%	32/46	69.6%	0.25*
Years since qualified	22	(14–27)	23	(17–27)	0.38†
Clinicians who recruited					
Number of recruiting clinicians	30/58	51.7%	34/46	73.9%	0.021*
Number of recruiting nurses	7/11	63.6%	11/13	84.6%	0.36‡
Number of recruiting GPs	23/47	48.9%	23/33	69.7%	0.064*
Number of children recruited by clinician type					
Number of children recruited by nurses	65/209	31.1%	155/292	53.1%	<0.001*
Number of children recruited by GPs	144/209	68.9%	137/292	46.9%	<0.001*
Recruitment of children					
Median number of children recruited per clinician	1	(0–4)	3	(0–11)	0.014†
Median number recruited per nurse	3	(0–7)	12	(3-17)	0.13†
Median number recruited per GP	0	(0–4)	2	(0–4)	0.077†

*χ^2^ test.

†Mann-Whitney U test.

‡Fisher’s exact test.

GP, general practitioner.

#### Demographics

There was no marked difference between arms with regard to gender, ethnicity or deprivation score (table 2). The median age of the children in the intervention group was younger compared with the control group (2 vs 3 years, p=0.03) and the intervention children lived in households with proportionally more smokers (29% vs 17% controls, p=0.002).

**Table 2:** Demographic profile in each arm of the trial

	Control		Intervention		
	Median	IQR	Median	IQR	p Value
Child age	3 years	1–5 years	2 years	1–4 years	0.028†
Home IMD* score	12.1	6.9–21.5	12.1	6.5–23.9	0.74†
	n/N	%	n/N	%	
Gender (male)	105/209	50.2%	139/292	47.6%	0.56‡
Any smoker in household	34/206	16.5%	82/288	28.5%	0.020‡
Ethnicity					
White	187/209	89.5%	251/292	86.0%	0.72 (4 df)‡
Mixed	8/209	3.8%	17/292	5.8%	
Asian or Asian British	7/209	3.4%	9/292	3.1%	
Black or black British	5/209	2.4%	11/292	3.8%	
Other ethnic group	2/209	1.0%	4/292	1.4%	

*Index of multiple deprivation.

†Mann-Whitney U test.

‡χ^2^ test.

IMD, Index of Multiple Deprivation.

#### Symptoms, signs and health outcomes

The majority of symptoms and signs were more severe among the intervention children (table 3) who had a longer illness duration prior to consultation (p=0.03), a higher respiratory rate (p<0.001), higher wheeze prevalence (p=0.007), higher global illness severity scores, measured by both the carer (p=0.045) and clinician (p=0.02) and children where the clinician had a ‘gut feeling’ that something was wrong (p=0.03). There was little evidence of a difference between days to cough resolution (median 14 days, IQR 9–25 days in intervention arm compared with a median of 13 days, IQR 8–27 days in the control arm, p=0.77) or follow-up appointments arranged during consultation (primary or secondary: 2.1% vs 1.0% controls, p=0.33). There was a slightly higher proportion of children in the intervention arm attending emergency departments in the 30 days postrecruitment (2.1% vs 0.5% controls, p=0.14), although none of these children received antibiotic treatment. There was one hospital admission in the 30 days postrecruitment; the child was in the intervention arm and was sent home the same day with a discharge diagnosis of ‘viral-induced wheeze’.

**Table 3:** Comparison of children’s symptoms and signs

	Control		Intervention		
	Median	(IQR)	Median	(IQR)	p Value
Symptom or sign					
Illness duration prior to consultation	5 days	(3–13)	7 days	(4–14)	0.034^†^
Clinician reported illness severity score (0–10)	3	(2–4)	3	(2–4)	0.022^†^
	Mean	SD	Mean	SD	
Carer reported illness severity score (0–10)	4.89	1.75	5.23	1.94	0.045^‡^
	n/N	**%**	n/N	**%**	§
Severe cough (24 hours prior to consultation)	41/208	19.7%	79/292	27.1%	0.058
Severe fever (24 hours prior to consultation)	15/208	7.2%	26/292	8.9%	0.50
Moderate or severe vomiting (24 hours prior)	18/208	8.7%	41/292	14.0%	0.066
High temperature (≥37.8^°^C)	29/208	13.9%	29/292	9.9%	0.17
High respiratory rate*	11/208	5.3%	44/292	15.1%	<0.001
Intersubcostal recession	5/208	2.4%	11/292	3.8%	0.39
Wheeze (auscultation)	17/208	8.2%	48/292	16.4%	0.007
Crackles or crepitations	37/208	17.8%	65/292	22.3%	0.22
Clinician had ‘gut feeling’ something was wrong	12/208	5.8%	34/292	11.6%	0.025

*Using age-related cut-offs.

†Mann-Whitney U test.

‡T-test.

§χ^2^ test.

#### Antibiotic prescribing

As table 4 shows, in the intervention group of the CHICO feasibility trial the overall antibiotic prescribing rate at consultation was 25% (19.9% immediate and 5.1% delayed)— demonstrating a marked reduction in both immediate and delayed prescribing compared with estimates from a few years earlier. However, the prescribing rate among the control children in the study was even lower (15.8%, 14.4% immediate and 1.4% delayed).

**Table 4:** Antibiotics prescribed at consultation in the two arms of the trial

Prescribing	Control		Intervention		
	N	%	N	%	p Value
No antibiotics prescribed	176/209	84.3	219/292	75.0	0.018 (2 df)
Immediate antibiotics prescribed	30/209	14.4	58/292	19.9	
Delayed antibiotics prescribed	3/209	1.4	15/292	5.1	

### Fidelity and acceptability of the intervention

None of the data collection web sessions during consultation was abandoned and no technical issues were reported with the website after resolving early teething problems associated with the use of older web-browsers. According to the available web page time-stamp data, the median time taken to complete the intervention was 5–6 min. This was consistent with clinicians reporting in the interviews that the intervention added 5–10 min onto their consultations. Some clinicians said they were reassured that hospitalisation risk agreed with their clinical judgement and some felt it was a useful backup for carers. The pages describing the background to the trial were only accessed on 29 occasions, although we are not able to discern whether these were unique visitors, or multiple visits from a few clinicians. Clinicians printed the personalised leaflet for the majority of the carers (92%), but only 4 of the 14 carers interviewed recalled receiving and reading the printout. No modifications were made to the intervention during the feasibility study.

### Qualitative interviews with clinicians and carers

Interviews were conducted with 28 clinicians (17 GPs and 11 nurses) and 14 carers sampled with varying characteristics (table 5). Selected quotes from the clinicians in each arm of the trial are listed in table 6.

**Table 5:** Description of clinicians and carers sampled for the qualitative analysis

	Characteristic	N	**%**
Clinician sample			
Total clinicians in sample		28	100%
Study arm	*Intervention*	16	57.1%
	*Control*	12	42.9%
Clinician type	*GP*	17	60.7%
	*Nurse*	11	39.3%
Clinician recruitment rate	*>20 children*	5	17.9%
	*10–20 children*	11	39.3%
	*<10 children*	12	42.9%
Clinician antibiotic	>25%	9	32.1%
Prescribing rate	15%–24%	9	32.1%
	<15%	10	35.7%
Carer sample			
Total carers in sample		14	100%
IMD quintile*	*1 (most deprived)*	1	7.1%
	*2*	5	35.7%
	*3*	2	14.3%
	*4*	3	21.4%
	*5 (most affluent)*	3	21.4%
Child age (in years)	*<2*	8	57.1%
	*2–4*	4	28.6%
	*5+*	2	14.3%
Hospitalisation risk	*Low risk—home care*	5	35.7%
Treatment decision	*Low risk*—*delayed antibiotics*	3	21.4%
	*Low risk*—*immediate antibiotics*	1	7.1%
	*Medium risk*—*home care*	3	21.4%
	*Medium risk*—*delayed antibiotics*	0	0.0%
	*Medium risk*—*immediate antibiotics*	2	14.3%

*IMD based on the postcode of the family home.

IMD, Index of Multiple Deprivation.

**Table 6:** Verbatim quotes from the clinicians

	Quotes (type of clinician, number, arm of trial)
Q1	‘*if they were quite poorly I wouldn’t be putting them into CHICO, because it would take me um… longer to do that consultation and examination and think about the plan of care.*’ (NP, #182, Intervention Arm)
Q2	‘*in a busy duty surgery, whilst triaging, would just forget and would book them in as the normal route through the duty surgery or with a nurse practitioner. So there were definitely probably children being seen that were missed*.’ (GP, #207, Control Arm)
Q3	‘*need to examine them first to make sure that they weren’t acutely unwell.*’ (GP, #145, Intervention Arm)
Q4	‘*generally by the time you’ve got to that (the odds ratios) you’ve given your advice, haven’t you, and you’ve got a gut instinct that actually there isn’t anything wrong with that child*.’ (NP, # 162, Intervention Arm)
Q5	‘*I would say that the question that skewed the algorithm the most, in my opinion, was the vomiting one. I think a lot of times parents would be over-reporting severity of vomiting. And that’s difficult, I know, because you’re looking for subjective data from parents*.’ (GP, #133, Intervention Arm)
Q6	‘*I don’t think it influenced me in my antibiotic prescribing at all. … I’m very aware that we over-prescribe, but I think that, you know, like I said, I think there are times where I overrode your system regardless*.’ (NP, #104, Intervention Arm)
Q7	‘*it was nice to have the reassurance that the algorithm backed me up, if I’d thought that they didn’t need antibiotics…. And sometimes I used that to reassure parents as well. But… I knew what I was going to do based on history and examination already, as opposed to using the algorithm to dictate my choices of what I was going to do*.’ (NP, #133 Intervention Arm)
Q8	*‘But there were some conflicting things where it said, ‘No antibiotics,’ and I was going to prescribe anyway. So I just said, ‘I know it says that, but actually I feel* (prescribing antibiotics) *is more appropriate,’ for whatever reasons.* (GP, #104, Intervention Arm)

NP, nurse practitioner; GP, general practitioner.

#### Recruitment

Carers perceived the data burden across the period of the study to be light. Clinicians, carers and children all liked the recruitment packs, which had been designed by a graphic designer, to be colourful and child-friendly. Clinicians in both arms reported recruiting less unwell children, for whom consultations were expected to be quicker to treat and therefore easier to combine with additional research activity (table 6: Q1). Although clinicians commented that the intervention was easy and quick to use compared with other studies, it still added time to the consultation. Practices with an appointment system that channelled eligible children towards active recruiters, who were often minor illness nurses, had high recruitment rates. In contrast, practices that normally channelled same-day appointments towards particular (non-recruiting) clinicians tended to be very low recruiters, despite attempts to get eligible patients redirected to recruiters (table 6: Q2).

#### Using the intervention

Some clinicians in both arms initiated recruitment only after they had completed their ‘normal consultation’. Most said this was because engaging with the web-based tool interfered with their ‘normal consultation’, others that they needed to assess the child for serious illness before deciding whether to include them in the trial (table 6: Q3). Clinicians reported a high degree of awareness of the problem of high antibiotic prescribing rates and described a range of other concurrent initiatives aimed at reducing antibiotic prescribing.

Intervention clinicians rarely reported using the CHICO algorithm results as a decision aid. Their reasons for this included: i) their ‘normal consultation’ including treatment decision was undertaken before initiating the intervention; ii) they decided early in the consultation that they did not need to prescribe and therefore extra information was not useful (table 6: Q4) (perhaps exacerbated where they chose not to recruit sicker children); iii) they lacked trust in the algorithm (table 6: Q5). Clinicians said the questions/information included in the intervention were more detailed than they usually asked, but none felt it had influenced or changed their decision. They were reassured when the reported risk of hospitalisation agreed with their clinical judgement, and could be used as a useful backup to present to carers (table 6: Q7). If the hospitalisation risk or advice provided was at odds with their clinical judgement, a few clinicians said that they ignored the advice (table 6: Q8).

Most carers said the CHICO consultation appeared as normal to them, while two said the history taking or explanations were more detailed. Few carers were aware of the reported risk of hospitalisation or subsequent advice from the CHICO intervention; one was reassured by the low-risk advice. Two had experiences of the clinician prescribing antibiotics despite CHICO advice.

#### The carer leaflet

Clinicians valued having the leaflet to print out for carers highly; some said it was a useful alternative to a prescription. Some felt it backed up their recommendations and others felt it was good for carers to have printed information. Carers had mixed views about the leaflet; some felt it did not tell them anything they did not know already; others said it gave them some useful information; and in one case the carer reported that it prevented a reconsultation.

### Health economics

The review of patient practice records and the online data collection system, supported by telephone calls to carers, provided a comprehensive source of information on the NHS resource use, although data missingness increased with symptom duration. Almost 99% of carers reported some NHS resource use. The initial consultation was estimated to take 15.29 min in the intervention arm compared with 10 min in the control arm. Health system cost data were positively skewed, reflecting limited NHS costs other than for the initial GP consultation.

Mean NHS costs per patient from available cases (n=494) were £54.62 in control practices, and £78.78 in intervention practices. The difference of £24.15 is associated with a bias corrected and accelerated 95% CI of £18.57 to £32.98. Mean per-patient costs were primarily determined by the costs of the initial consultation, which constitute 73% of the NHS costs in the control arm and 78% in the intervention arm.

Only one in four recruited children were in the age range of 5–11 suitable for the CHU-9D instrument; moreover, only 44 (9% of all participants) provided baseline and follow-up data from the week of resolution to facilitate the estimation of quality-adjusted life-years. The data on these children tentatively suggest that their quality of life returned to population norms after the baseline appointment, although this finding must be interpreted cautiously in light of the limited availability of these data.

## Discussion

### Summary of main results

The feasibility study showed high recruitment and retention. Quantitative and qualitative data confirm the intervention was acceptable to the clinicians and widely used, but often postconsultation and not within-consultation as intended. We found evidence of postrandomisation differential recruitment, which is likely to have biased our estimates of intervention effect and explain our paradoxical results.

Evidence for differential recruitment included children in the intervention arm having more severe baseline characteristics than control children and higher recruitment rates in the intervention than control arm.

In the qualitative interviews, clinicians from both arms of the trial reported preferential recruitment of less unwell children as these were quicker to deal with and easier to combine with the research. This was particularly true in the control arm, where larger numbers of well children, relative to the intervention arm, were recruited. Evidencing Hawthorne effects (whereby clinicians modified their behaviour in response to their awareness of being in the trial) is challenging in RCTs without concurrent observational studies, but our 2011–2013 cohort study (in which we developed the algorithm)^19^ showed antibiotic prescribing rates (37%) higher than both our intervention (25%) and control (16%) arms. It is therefore possible that Hawthorne effects may have been acting to reduce prescribing in both arms of our trial. Although we did not overtly advertise that the trial was focussing on reducing antibiotic prescribing, the clinicians may have inferred the aims of the trial. Our previous work with these practices may have alerted some clinicians to our aims, coupled with national and local campaigns to increase awareness of antimicrobial resistance. The control clinicians used the same web-based tool during consultation to record study data. Thus, our data collection in itself among the controls may have inadvertently increased risk perception (by providing risk information, although partially obscured among the data collected that was previously unavailable to clinicians) as well as provide ‘test’ conditions.

The web-based data collection tool and review of medical notes were appropriate vehicles for resource-use data collection. The principal driver of higher NHS costs in the intervention group was longer initial GP appointments, when the web-based system was being used. There was little evidence on quality of life because of limited data availability.

### Strengths and weaknesses

There are many strengths to this feasibility trial.

- Recruitment was so rapid that we stopped a month earlier than planned, with robust data collection and few missing values.
- We adequately resourced the trial to achieve excellent follow-up with little attrition, building up relationships with the families and using several types of communication media as an effective way of obtaining data for our web-based entry system.
- We reduced variation in practice recruitment levels, compared with our previous cohort study,^19^ with a higher median number of patients per practice and fewer non-recruiting practices.
- None of the intervention web-sessions was abandoned and clinicians thought it was quick and easy to use compared with other interventions.
- Clinicians highly valued having the personalised leaflet as an alternative to a prescription.

Apart from differential recruitment and potential Hawthorne effects, we are aware of three other weaknesses.

- First, the intervention was designed to be used as part of a decision aid, but it became apparent that some clinicians only used the intervention after the treatment decision was made. The intervention might be more acceptable and easier to use if embedded within the existing practice records system.
- Second, using the intervention added around five minutes to consultation time, which could be reduced in future. Some of this time was to record research data and consideration needs to be given as to whether these data can be collected outside the consultation either from the medical notes or at practice level.
- Finally, although clinicians liked the personalised leaflets, carers were less enthusiastic. Further development through additional parent consultation would be recommended for a future trial.

### Results in context with other research

We have constructed a complex intervention associated with lower antibiotic prescribing rates (from 37% to 25%) compared with data from similar practices in the same area a couple of years earlier,^19^ but an even lower rate among concurrent controls (16%). Local data over a 4-year period suggest the extremely low prescribing rates among the control practices do not reflect usual practice, which may be due to differential recruitment.

Antibiotic prescription data from a large primary care database, covering 537 UK general practices during 1995–2011 suggest the rate of prescribing for RTIs in children and adults initially fell around the millennium but by 2011 had risen to 40%.^28^ Our estimate of 37% among children from a large cohort study conducted between 2011 and 2013^19^ is a robust estimate and a reduction in prescribing by almost a third to 25% is worth pursuing. To do this, a future trial must overcome the dual problems of differential recruitment and Hawthorne effects.

A primary care feasibility trial of patients with back pain^29^ that also experienced differential recruitment recommended identifying patients, using independent researchers, before randomisation. However, back pain is a chronic condition giving research teams time to identify and invite patient participation. The acute nature of childhood respiratory infections prevent prior identification and the cost of using independent researchers at multiple primary care sites would be prohibitive. One approach would be to select primary care sites that adopt a triage system for children with cough and work more closely with these different systems to help reduce selection bias. An alternative would be a ‘lighter touch’ design using practice level using routinely collected data and practice level consent, negating the need to consent and recruit individual patients postrandomisation. The unit of interest would be the practice rather than the patient, so all patients seen in a specified time would be included. Amoxicillin suspension is the main antibiotic given to children with acute cough and RTI and has been successfully used previously^30^ as a proxy marker to assess antibiotic prescribing at the practice level.

The Hawthorne effect is not uncommon in clinical trials in general or indeed in trials specifically aimed at reducing antibiotic prescribing,^31^ but the mechanisms of effect and magnitude are not well understood.^32^

## Conclusions

Many valuable lessons have been learnt from this feasibility study, and a redesign is required for any future trial. To negate differential recruitment and reduce the possibility of a Hawthorne effect, a ‘light touch’ efficient design is needed that avoids patient recruitment at the clinician level and uses data already routinely collected by the practices themselves. Better training in the use of the intervention and encouragement to use the tool as part of the consultation process would be facilitated if the intervention is embedded within current practice systems. Removing the need for clinicians to recruit patients or enter data other than that required for the tool would both reduce the time added to consultations and preserve usual practice among the control clinicians.

10.1136/bmjopen-2016-014506.supp1 Supplementary data 1

10.1136/bmjopen-2016-014506.supp2 Supplementary data 2

## Supplementary Material

Reviewer comments

Author's manuscript
