# Supplementary material for: Feasibility cluster randomised controlled trial of a within-consultation intervention to reduce antibiotic prescribing for children presenting to primary care with acute respiratory tract infection and cough
Source: BMJ Open. 2017 May 9;7(5):e014506. doi: 10.1136/bmjopen-2016-014506 (PMC5623421; doi:10.1136/bmjopen-2016-014506)
Supplement: Supplementary data 1 [file bmjopen-2016-014506supp001.pdf]

# COREQ Statement

**Feasibility cluster randomised controlled trial of a within-consultation intervention to reduce antibiotic prescribing for children presenting to primary care with acute respiratory tract infection and cough**

## Domain 1: Research team and reflexivity

*Personal Characteristics*

1. Interviewer/facilitator. Which author/s conducted the interview or focus group?
Dr Christie Cabral

2. Credentials. What were the researcher's credentials? E.g. PhD, MD.
*PhD*

3. Occupation. What was their occupation at the time of the study?
*Research Fellow*

4. Gender. Was the researcher male or female?
*Female.*

5. Experience and training. What experience or training did the researcher have?
*Extensive training in qualitative research methods and XX years' experience conducting qualitative research.*

*Relationship with participants*

6. Relationship established. Was a relationship established prior to study commencement?
*No*

7. Participant knowledge of the interviewer. What did the participants know about the researcher? e.g. personal goals, reasons for doing the research?
*The personal goals of the researcher were to complete the aims and objectives of the study only. The researcher had no personal goals or reasons for doing the research. As part of recruitment and gaining informed consent clinicians were fully informed about the aims and objectives of the study.*

8. Interviewer characteristics. What characteristics were reported about the interviewer/facilitator? e.g. Bias, assumptions, reasons and interests in the research topic.
*The interviewer (Christie Cabral) is a social scientist. Christie declared her academic standpoint to interviewees so that they were aware she was not from a medical background. We found this to be beneficial as Christie was able to ask 'naïve' questions and ask for clarification where needed – this produced some rich data and minimized bias.*

## Domain 2: study design

*Theoretical framework*

9. Methodological orientation and Theory. What methodological orientation was stated to underpin the study? e.g. grounded theory, discourse analysis, ethnography, phenomenology, content analysis
*In the Data Analysis section we explain how we used semi-structured interviews and thematic analysis, this was informed by grounded theory and the constant comparison method.*

*Participant selection*

10. Sampling. How were participants selected? e.g. purposive, convenience, consecutive, snowball.

*Purposive sampling was used to select interviewees in order to attempt to capture maximum variation in views and experiences in order that they adequately reflect those of a range of carers and clinicians involved in the trial. For carers from the intervention arm, a purposive sample was drawn in relation to child age, home neighbourhood social-economic deprivation, illness severity scores, and treatment outcomes. For clinicians from both trial arms a purposive sample was drawn in relation to, study arm, recruitment rate, antibiotic prescribing rate and professional role.*

11. Method of approach. How were participants approached? e.g. face-to-face, telephone, mail, email

*Parents who had provided consent to be contacted for a qualitative interview were contacted by telephone at preferred time. Clinicians who had agreed to take part in a qualitative interview were contacted by email with a follow up phone call if they did not respond to email.*

12. Sample size. How many participants were in the study?

*We recruited 28 clinicians and 14 carers*

13. Non-participation. How many people refused to participate or dropped out? Reasons

*Of those invited to participate in an interview, 4 clinicians (2 from each arm) and 3 parents did not complete an interview. Clinicians gave being too busy as their reason for not participating. Parents simply did not respond to further attempts to contact them, so it was not possible to record their reason for no participating.*

*Setting*

14. Setting of data collection. Where was the data collected? e.g. home, clinic, workplace

*Interviews were conducted over the telephone.*

15. Presence of non-participants. Was anyone else present besides the participants and researchers?

*No*

16. Description of sample. What are the important characteristics of the sample? e.g. demographic data, date.

*Table 5 outlines a description of clinicians and carers sampled for the qualitative analysis. We interviewed 28 clinicians (17 GP's and 11 nurses) with 57% from the intervention arm. We interviewed 14 carers from the intervention arm selected from areas of high and low social-economic deprivation, who consulted for children with ranging in ages (8 children <2 years, 4 children between 2-4 years and 2 over 5 years) and had a range of treatment decisions.*

*Data collection*

17. Interview guide. Were questions, prompts, guides provided by the authors? Was it pilot tested?

*A semi-structured, topic guide used for all interviews. The focus of the topic guide was participants' views and experiences of the trial. The topic guide was used flexibly so that interviewees could raise other issues they felt were important and also allowed for in-depth exploration of emergent themes.*

18. Repeat interviews.

*We did not conduct repeat interviews*

19. Audio/visual recording. Did the research use audio or visual recording to collect the data?

*Yes audio recording was used throughout data collection.*

20. Field notes. Were field notes made during and/or after the interview or focus group?
*Brief field notes were made after the interviews.*

21. Duration. What was the duration of the interviews or focus group?
*Between 10 and 43 minutes*

22. Data saturation. Was data saturation discussed?
*Yes this was discussed in team meetings.*

23. Transcripts returned. Were transcripts returned to participants for comment and/or correction?
*No. We did not feel this was possible to offer in the time available in the study.*

**Domain 3: analysis and findings**
*Data analysis*
24. Number of data coders. How many data coders coded the data?
*Data was initially coded by the researcher Christie Cabral. Jeremy Horwood, a senior qualitative research fellow double coded a subset of 10% of the transcripts to inform the coding framework and ensure robust analysis. Interpretation of data was discussed regularly at multidisciplinary team meetings.*

25. Description of the coding tree. Did authors provide a description of the coding tree?
*No.*

26. Derivation of themes. Were themes identified in advance or derived from the data?
*Themes were derived inductively from the data.*

27. Software. What software, if applicable, was used to manage the data?
*We used NVivo 10 qualitative software package to manage the data.*

28. Participant checking. Did participants provide feedback on the findings?
*No.*

29. Quotations presented. Were participant quotations presented to illustrate the themes / findings? Was each quotation identified? e.g. participant number
Yes, quotations to illustrate key themes are presented in Table 6.

30. Data and findings consistent. Was there consistency between the data presented and the findings?
*Yes, we think so.*

31. Clarity of major themes. Were major themes clearly presented in the findings?
*Yes, we think so.*

32. Clarity of minor themes. Is there a description of diverse cases or discussion of minor themes?
*Yes, we think so.*
