# Supplementary material for: Feasibility cluster randomised controlled trial of a within-consultation intervention to reduce antibiotic prescribing for children presenting to primary care with acute respiratory tract infection and cough
Source: BMJ Open. 2017 May 9;7(5):e014506. doi: 10.1136/bmjopen-2016-014506 (PMC5623421; doi:10.1136/bmjopen-2016-014506)
Supplement: Supplementary data 2 [file bmjopen-2016-014506supp002.pdf]

Table 1: CONSORT 2010 checklist of information to include when reporting a cluster randomised trial

Section/Topic	Item No	Standard Checklist item	Extension for cluster designs	Page No *
**Title and abstract**				
	1a	Identification as a randomised trial in the title	Identification as a cluster randomised trial in the title	Yes (page 1)
	1b	Structured summary of trial design, methods, results, and conclusions (for specific guidance see CONSORT for abstracts)[1,2]	See table 2	Yes (page 2)
**Introduction**				
**Background and objectives**	2a	Scientific background and explanation of rationale	Rationale for using a cluster design	Yes (page 4). For this type of knowledge intervention trial clustering is self evident so explanation not provided (given the word limit)
	2b	Specific objectives or hypotheses	Whether objectives pertain to the the cluster level, the individual participant level or both	Yes (page 4) although this is a feasibility study so the main objectives surround issues of recruitment and acceptability of the intervention
**Methods**				
**Trial design**	3a	Description of trial design (such as parallel, factorial) including allocation ratio	Definition of cluster and description of how the design features apply to the clusters	Yes (page 5)
	3b	Important changes to methods after trial commencement (such as eligibility criteria), with reasons		No important changes made
**Participants**	4a	Eligibility criteria for	Eligibility criteria for clusters	Yes (page 5)

		participants		
	4b	Settings and locations where the data were collected		Yes (page 5)
**Interventions**	5	The interventions for each group with sufficient details to allow replication, including how and when they were actually administered	Whether interventions pertain to the cluster level, the individual participant level or both	Yes (page 5)
**Outcomes**	6a	Completely defined pre-specified primary and secondary outcome measures, including how and when they were assessed	Whether outcome measures pertain to the cluster level, the individual participant level or both	Yes (pages 6 and 7) although these refer specifically to the feasibility study
	6b	Any changes to trial outcomes after the trial commenced, with reasons		No changes made
**Sample size**	7a	How sample size was determined	Method of calculation, number of clusters(s) (and whether equal or unequal cluster sizes are assumed), cluster size, a coefficient of intracluster correlation (ICC or *k*), and an indication of its uncertainty	This was a feasibility trial so no formal sample size required although our pragmatic approach has been described (page 5)
	7b	When applicable, explanation of any interim analyses and stopping guidelines		Not applicable
**Randomisation:**				
**Sequence generation**	8a	Method used to generate the random allocation sequence		Yes (page 5)
	8b	Type of randomisation; details of any restriction (such as blocking and block size)	Details of stratification or matching if used	Yes (page 5)
**Allocation concealment mechanism**	9	Mechanism used to implement the random allocation sequence (such as sequentially numbered containers), describing any	Specification that allocation was based on clusters rather than individuals and whether allocation concealment (if any) was at the cluster level, the individual	Yes (page 5) Simple randomisation used at cluster level, a one-off

		steps taken to conceal the sequence until interventions were assigned	participant level or both	procedure
**Implementation**	10	Who generated the random allocation sequence, who enrolled participants, and who assigned participants to interventions	Replace by 10a, 10b and 10c	
	10a		Who generated the random allocation sequence, who enrolled clusters, and who assigned clusters to interventions	Yes (page 5)
	10b		Mechanism by which individual participants were included in clusters for the purposes of the trial (such as complete enumeration, random sampling)	Yes (page 5)
	10c		From whom consent was sought (representatives of the cluster, or individual cluster members, or both), and whether consent was sought before or after randomisation	Yes (page 5)
**Blinding**	11a	If done, who was blinded after assignment to interventions (for example, participants, care providers, those assessing outcomes) and how		Not applicable (blinding could not be used)
	11b	If relevant, description of the similarity of interventions		Not applicable
**Statistical methods**	12a	Statistical methods used to compare groups for primary and secondary outcomes	How clustering was taken into account	Yes (page 7)
	12b	Methods for additional analyses, such as subgroup analyses and adjusted analyses		Yes (page 7)
**Results**				

**Participant flow (a diagram is strongly recommended)**	13a	For each group, the numbers of participants who were randomly assigned, received intended treatment, and were analysed for the primary outcome	For each group, the numbers of clusters that were randomly assigned, received intended treatment, and were analysed for the primary outcome	Yes (page 8)
	13b	For each group, losses and exclusions after randomisation, together with reasons	For each group, losses and exclusions for both clusters and individual cluster members	Yes (page 8)
**Recruitment**	14a	Dates defining the periods of recruitment and follow-up		Yes (page 8)
	14b	Why the trial ended or was stopped		Not applicable
**Baseline data**	15	A table showing baseline demographic and clinical characteristics for each group	Baseline characteristics for the individual and cluster levels as applicable for each group	Yes (page 20)
**Numbers analysed**	16	For each group, number of participants (denominator) included in each analysis and whether the analysis was by original assigned groups	For each group, number of clusters included in each analysis	Yes (page 8 and page 20)
**Outcomes and estimation**	17a	For each primary and secondary outcome, results for each group, and the estimated effect size and its precision (such as 95% confidence interval)	Results at the individual or cluster level as applicable and a coefficient of intracluster correlation (ICC or k) for each primary outcome	Yes (page 21) but only to a point as this is a feasibility trial
	17b	For binary outcomes, presentation of both absolute and relative effect sizes is recommended		Yes (page 21)
**Ancillary analyses**	18	Results of any other analyses performed, including subgroup analyses and adjusted analyses, distinguishing pre-specified from exploratory		Not applicable, the main analysis surrounds recruitment issues and qualitative work as part of a feasibility study
**Harms**	19	All important harms or unintended effects in each		No adverse events to report

		group (for specific guidance see CONSORT for harms[3])		
**Discussion**				
**Limitations**	20	Trial limitations, addressing sources of potential bias, imprecision, and, if relevant, multiplicity of analyses		Yes (pages 12 and 13) and summarised as requested (page 3)
**Generalisability**	21	Generalisability (external validity, applicability) of the trial findings	Generalisability to clusters and/or individual participants (as relevant)	Yes (pages 13 and 14)
**Interpretation**	22	Interpretation consistent with results, balancing benefits and harms, and considering other relevant evidence		Yes (pages 13 and 14)
**Other information**				
**Registration**	23	Registration number and name of trial registry		Yes (page 15)
**Protocol**	24	Where the full trial protocol can be accessed, if available		The trial protocol paper is referenced (page 5)
**Funding**	25	Sources of funding and other support (such as supply of drugs), role of funders		Yes (page 15)

** Note: page numbers optional depending on journal requirements*

[1] Hopewell S, Clarke M, Moher D, Wager E, Middleton P, Altman DG, et al. CONSORT for reporting randomised trials in journal and conference abstracts. *Lancet* 2008, 371:281-283

[2] Hopewell S, Clarke M, Moher D, Wager E, Middleton P, Altman DG at al (2008) CONSORT for reporting randomized controlled trials in journal and conference abstracts: explanation and elaboration. *PLoS Med* 5(1): e20

[3] Ioannidis JP, Evans SJ, Gotzsche PC, O'Neill RT, Altman DG, Schulz K, Moher D. Better reporting of harms in randomized trials: an extension of the CONSORT statement. *Ann Intern Med* 2004; 141(10):781-788.
