# Supplementary material for: Feasibility cluster randomised controlled trial of a within-consultation intervention to reduce antibiotic prescribing for children presenting to primary care with acute respiratory tract infection and cough
Source: BMJ Open. 2017 May 9;7(5):e014506. doi: 10.1136/bmjopen-2016-014506 (PMC5623421; doi:10.1136/bmjopen-2016-014506)
Supplement: Author's manuscript [file bmjopen-2016-014506.draft_revisions.pdf]

BMJ Open

Feasibility cluster randomised controlled trial of a within-consultation intervention to reduce antibiotic prescribing for children presenting to primary care with acute respiratory tract infection and cough

Journal:	*BMJ Open*
Manuscript ID	bmjopen-2016-014506
Article Type:	Research
Date Submitted by the Author:	29-Sep-2016

Complete List of Authors:	Blair, Peter; University of Bristol, School Of Social & Community Medicine<br>Turnbull, Sophie; University of Bristol, School of Social and Community Medicine<br>Ingram, Jenny; Bristol University , School of Social & Community Medicine<br>Redmond, Niamh; University of Bristol, Academic Unit of Primary Health Care<br>Lucas, Patricia; Unversity of Bristol, School for Policy Studies<br>Cabral, Christie; University of Bristol, School of Social and Community Medicine<br>Hollinghurst, Sandra; University of Bristol, Community Based Medicine<br>Dixon, Padraig; University of Bristol, School of Social and Community Medicine, University of Bristol; School of Social and Community Medicine<br>Peters, Tim; University of Bristol, Department of Community Based Medicine<br>Horwood, Jeremy; University of Bristol, UK, School of Social and Community Medicine<br>Little, Paul; University of Southampton, Primary Care and Population Science;<br>Francis, Nick A.; Cardiff University School of Medicine, School of Medicine<br>Gilbertson, Anna; University of Bristol, School Of Social & Community Medicine<br>Jameson, Catherine; University of Bristol, School Of Social & Community Medicine<br>Hay, Alastair; University of Bristol, Academic Unit of Primary Health Care

**Primary Subject Heading**:	General practice / Family practice
Secondary Subject Heading:	Respiratory medicine, Infectious diseases
Keywords:	Cough, Antibiotics, Respiratory Tract Infections (RTIs), PRIMARY CARE, Children, Feasibility

SCHOLARONE™ Manuscripts

For peer review only

Feasibility cluster randomised controlled trial of a within-consultation intervention to reduce antibiotic prescribing for children presenting to primary care with acute respiratory tract infection and cough

Blair PS[1], Turnbull S[2], Ingram JC[1], Redmond NM[2,3], Lucas PJ[4], Cabral C[2], Hollinghurst S[2,3], Dixon P[5], Peters TJ[3,6], Horwood J[2,3], Little P[7], Francis N[8], Gilbertson A[2], Jameson C[2], Hay AD[2].

[1] Centre for Child and Adolescent Health, School of Social and Community Medicine, University of Bristol UK.

[2] Centre for Academic Primary Care, School of Social and Community Medicine, University of Bristol, UK

[3] National Institute for Health Research Collaborations for Leadership in Applied Health Research and Care West (NIHR CLAHRC West), University Hospitals Bristol NHS Foundation Trust, UK

[4] School for Policy Studies, University of Bristol, UK

[5] School of Social and Community Medicine, University of Bristol, UK

[6] School of Clinical Sciences, University of Bristol, UK

[7] Department of Primary Care & Population Sciences, University of Southampton, UK

[8] Division of Population Medicine, Cardiff University, UK

Corresponding author

Dr Peter S Blair, Reader in Medical Statistics
Level D, St Michael's Hospital, Southwell St, Bristol, BS2 8EG.
Phone: 01173425145
Fax: 01173425154
Email: p.s.blair@bris.ac.uk

Trial registration: ISRCTN23547970

UKCRN study ID: 16891

Keywords: Randomised controlled trial (RCT), Feasibility, Children, Respiratory tract infections (RTIs), Primary care, Cough, Antibiotics (or Anti-microbial agents)

Main paper Word count: 4313 words

For peer review only

Abstract

Objective: To investigate recruitment and retention, data collection methods and the acceptability of a 'within-consultation' complex intervention designed to reduce antibiotic prescribing.

Design: Primary care feasibility cluster randomised controlled trial.

Setting: 32 general practices in South West England recruiting children from October 2014 to April 2015.

Participants: Children (aged 3 months to <12 years) with acute cough and respiratory tract infection (RTI).

Intervention: A web-based clinician-focussed clinical rule to predict risk of future hospitalisation and a printed leaflet with individualised child health information for carers, safety-netting advice and a treatment decision record.

Controls: Usual practice, with clinicians recording data on symptoms, signs, and treatment decisions.

Results: Of 542 children invited, 501 (92.4%) consented to participate, a month ahead of schedule. Antibiotic prescribing data were collected for all children, follow-up data for 495 (98.8%) and NHS resource use data for 494 (98.6%). The overall antibiotic prescribing rates for children's RTIs were 25% and 15.8% (p=0.018) in intervention and control groups respectively. We found evidence of post-randomisation differential recruitment: the number of children recruited to the intervention arm was higher (292 vs. 209); over half were recruited by prescribing nurses compared with less than a third in the control arm; children in the intervention arm were younger (median age 2 vs. 3 years controls, p=0.03) and appeared to be more unwell than those in the control arm with higher respiratory rates (p<0.0001), wheeze prevalence (p=0.007) and illness severity scores assessed by both carers (p=0.045) and clinicians (p=0.01). Interviews with clinicians confirmed preferential recruitment of less unwell children to the trial, and more so in the control arm.

Conclusion: The observed differential recruitment may explain the paradoxical antibiotic prescribing rates. Future studies should consider a design which removes the need for individual consent post-randomisation and embeds the intervention within electronic primary care records.

Trial registration number: ISRCTN 23547970

Abstract Word count: 300/300 words

For peer review only

Strengths & Limitations

- Recruitment was successful with robust data collection and few missing values.
- We adequately resourced the trial to achieve excellent follow-up with little attrition,
- We reduced variation in practice recruitment levels, compared to our previous cohort study. .
- None of the intervention web-sessions were abandoned and clinicians thought it was quick and easy to use compared to other interventions.
- Clinicians also highly valued having the personalised leaflet as an alternative to a prescription although carers were less enthusiastic.
- The intervention was designed to be used as part of a decision aid, but it became apparent that some clinicians only used the intervention after the treatment decision was made.
- Using the intervention added around five minutes to consultation time. Some of this time was to record research data and consideration needs to be given as to whether these data can be collected outside the consultation.
- The intervention was a stand-alone system and might be more acceptable and easier to use if embedded within the existing practice records system.
- Differential recruitment was quite marked, in particular the intervention children were significantly more unwell. A future trial design needs to minimise or avoid this post-randomisation recruitment bias.
- The control clinicians used the same web-based tool during consultation to record study data. Thus our data collection in itself amongst the controls may have inadvertently increased risk perception as well as provide 'test' conditions to encourage a Hawthorne effect.

For peer review only

Introduction

Respiratory tract infections (RTIs) in children are extremely common and costly to service providers, families and employers in the UK.[1,2] Clinicians often prescribe antibiotics "just in case", if they feel uncertain about patient health or legal outcomes.[3] Clinical uncertainty in primary care regarding the diagnosis and best management of RTIs has led to the inappropriate use of existing antibiotics, which, combined with the slowing in development of new antibiotics, is associated with antimicrobial resistance.[4-6] This has been described as one of the greatest challenges to public health today,[7,8] with the World Economic Forum placing antibiotic-resistant bacteria on the 2014 Global Risks List.[9] The five-year NIHR-funded TARGET programme was set up in 2010 to derive new knowledge to improve the management of antibiotics given to children presenting to primary care with RTIs and cough.

Systematic reviews from this programme [10,11] found that passive strategies targeting only parents, such as waiting room posters or pamphlets, do not appear to alter prescribing rates significantly. The most effective interventions involved targeting both parents and clinicians during a consultation, using automatic computer prompts for evidence-based prescribing, involving clinicians in the design of the intervention, using printed materials with actionable information and integrating interventions into routine clinical processes. Qualitative research found that communication within consultations often failed to meet parents' needs: information on symptom relief was lacking, safety-netting advice was too vague to be useful and parent concerns went unaddressed.[3,12-15] Clinicians revealed that prognostic uncertainty is an important driver of antibiotic prescribing, and would like information to help them identify the children at risk of illness deterioration.[3,16,17]

Using a large multi-centre, prospective cohort study (over 8,300 children) we derived and internally validated a prognostic rule, using symptoms, signs, and demographic characteristics to predict hospitalisation in children presenting to primary care with acute cough and RTI.[18,19] Findings from across the TARGET programme were synthesised to develop a complex intervention, designed to reduce antibiotic prescribing. The CHICO (Children's Cough) feasibility trial reported here is the final element of the TARGET programme, the aim of which was to assess the recruitment and acceptability of the complex intervention and to understand whether it is feasible to conduct a larger trial.

For peer review only - http://bmjopen.bmj.com/site/about/guidelines.xhtml

Methods

Design

The CHICO feasibility trial was a primary care cluster randomised controlled trial (RCT) comparing a web-based intervention with usual care of children presenting to general practices with RTI and acute cough. The trial was approved by the North West-Haydock Research Ethics Committee, UK (reference number: 14/NW/1034, Trial registration: ISRCTN23547970, UKCRN study ID: 16891). The trial protocol[20] was devised according to the SPIRIT guidelines[21] for randomised controlled trials, reporting of the findings followed CONSORT guidelines for clustered trials[22] and below is a brief summary of the methodology.

Practice and patient recruitment

General practices were invited to participate in the trial from locations in Bristol and the surrounding areas; the only exclusion criterion was for those few practices using very outdated internet browsers. General Practitioners (GPs) and prescribing nurses (hereafter termed 'clinicians') were eligible to recruit children to the trial and informed consent was obtained from the parent or legal guardian (carers). Children were included if they were aged between 3 months and under 12 years and presenting with a RTI with acute cough of no more than 28 days duration prior to consultation. They were eligible if they presented with illnesses such as asthma (including those with infective exacerbations), epilepsy or diabetes. Children who required same day hospital assessment or admission were also included. Children were excluded if they were considered to have a high risk of serious infection (immune-compromised, cystic fibrosis, splenectomy).

Randomisation and sample size

The allocation process was a one-off randomisation stratified for both practice size and prevalence of antibiotic prescribing using a proxy measure of amoxicillin suspension prescriptions (the main antibiotic used for children with cough) within the previous 12 months. As this was a feasibility trial, a formal (effectiveness-based) sample size calculation was not required, although we were interested in the recruitment rate to inform a larger trial design. Recruitment rates varied widely between practices in our previous cohort study[19] so we monitored recruitment levels closely and capped recruitment at no more than 30 patients per practice. Estimates from the cohort study suggested 15 practices in each arm of the trial would yield a pragmatic sample between 300 and 500 patients over a seven-month period.

Intervention

A within-consultation interactive web-based tool was used both for data collection, and to deliver the following intervention elements:

For peer review only - http://bmjopen.bmj.com/site/about/guidelines.xhtml

1. Recording of patient's symptoms and signs.

2. Elicitation and recording carer concerns.

3. For each consultation children were identified as being at very low, average or higher than average risk of hospitalisation; current NICE guidelines on antibiotic use were also provided.

4. A personalised printout was produced for carers of each child. Clinical observations and carer concerns recorded in the system were used to produce this personalised information leaflet that explained the best home-care strategies and reinforced important safety netting advice. The aim of this leaflet was to give clinicians a tangible treatment action other than (or in addition to) prescribing, to improve safety-netting information and to provide home-care support for carers.

<u>Data collection</u>

Both control and intervention clinicians used the same web-based tool to obtain informed carer consent, enter baseline and symptom data during the consultation and record their actions taken. Additionally intervention clinicians were provided with background information about how the risk algorithm was developed, recorded carer concerns, were given on-screen the hospitalisation risk of the child and provided carers with a personalised printout. Use of the intervention was assessed by recording the number of times the clinicians used the web-based intervention and time spent on each page of the website. Follow-up data were collected each week from carers until the child's cough had resolved, or up to 8 weeks if not resolved. A medical records notes review was conducted at the recruiting practices to collect data relating to the 30 days following the recruitment consultation, including recruiting consultation timings, RTI-related antibiotic prescriptions, re-consultations, and hospitalisations.

<u>Qualitative Interviews</u>

Clinicians from both arms and carers from the intervention arm only were invited to participate in semi-structured interviews to explore their views of web-based data collection and the intervention. Interviews with carers were conducted either in the week following recruitment (to facilitate recall of the consultation) or after their child had recovered, to reflect on the whole follow-up data collection process. Purposive sampling was used to maximise variation in the sample of carers, including those with a range of child ages, home neighbourhood social-economic deprivation, illness severity scores, and treatment outcomes. Clinicians were purposefully sampled to include variation in recruiting and prescribing levels.

<u>Health Economics</u>

The purpose of the economic analysis was to inform the feasibility and design of a within-trial economic evaluation alongside a larger RCT, including assessing web-based data collection as a means of gathering comprehensive resource use and quality of life data from carers. Resources used

For peer review only - http://bmjopen.bmj.com/site/about/guidelines.xhtml

from the time of recruitment until the earliest of either resolution or the end of the eighth week of follow-up including: use of NHS resources (out-of-hours services, NHS 111, walk-in centres, and hospital and ambulance use), personal out-of-pocket expenditure such as over-the-counter medications and travel costs, and time off work. We examined quality of life using the Child Health Utility 9D (CHU-9D) instrument, a generic measure of paediatric quality of life validated for children aged 5-11 years. CHU-9D was included in the web-based tool for those children within the valid age-bracket.

Carers were given the option of providing data using a paper version rather than online if preferred. Participants were sent text and email reminders followed by telephone calls if the data were not returned promptly.[23,24] The length of the initial consultation was recorded electronically by the web-based tool and GP practice systems.

Data Analysis

Descriptive statistics were used to illustrate the characteristics of the carers, children and recruiting clinicians. For between-arm comparisons, categorical data were tested using Chi-square or Fisher's Exact test if an expected cell value was <5, continuous data were plotted and, if not normally distributed, non-parametric tests (Mann Whitney) were used. The use of baseline statistical tests to compare the arms is legitimate in the context of stratified cluster randomisation with subsequent (unconcealed) recruitment of individual participants. Such tests were used to ascertain if there was any evidence of differential recruitment of individuals across the trial arms beyond what would be expected by chance.

Qualitative interviews were audio-recorded, transcribed, anonymised and imported into NVIVO 10. Analysis of qualitative data began shortly after data collection started and was ongoing and iterative. An inductive thematic analysis approach was used to identify patterns and themes.[25] An initial coding frame was developed and refinded as new data were produced. Two researchers (CC and JH) double coded a subset of transcripts to inform the coding framework and ensure robust analysis.[26]

For both cost and quality of life data, the extent and nature of missing data was examined and compared between intervention and control practices. Descriptive statistics were used to explore resource use and cost by category and by arm to identify important cost drivers.

For peer review only - http://bmjopen.bmj.com/site/about/guidelines.xhtml

RESULTS

Ascertainment and data collection

A total of 32 practices were recruited to the trial, 16 randomised to each arm. Within the practices 104 clinicians signed up as recruiters and 64 recruited at least one child (18 nurses and 46 GPs). The target of 500 recruited children was achieved one month ahead of schedule (October 2014 to March 2015 inclusive); 542 children were invited to enter the trial and 501 agreed. Of the 41 who were not retained, 18 (3.3%) declined, 19 (3.5%) did not meet the eligibility criteria and 2 (0.4%) were subsequently withdrawn. We obtained complete carer-reported data for 495 (98.8%) from the 501 children retained in the trial. Only two practices recruited no children; the median number of children recruited at each practices was 16 (range 0-30, IQR 4-29). Primary care medical records were collected for 100% of the children. Web-based tool information was available for all but one of the 501 children.

Sample description

The median age of the 501 children in the trial was 2 years (IQR: 1-4 years, full range: 3 months-11 years), 49% were boys and there was no evidence of a difference in ethnicity when compared with 2011 UK Census data nor with the prevalence of smoking in the household when compared with a 2013 Opinions and Lifestyle survey. However, using the postcode of the family residence, the median IMD score in the trial (12.1) was markedly lower than national data (2010 Government statistics), suggesting the children in this cohort were less deprived than children in the general population.

Comparison between study arms

Recruitment

Although there were fewer clinicians in the intervention arm (46 vs. 58 controls) they recruited 40% more children (292 vs. 209 children). Table 1 shows a lower proportion of control group clinicians recruited children (p=0.02). In the intervention arm a higher proportion of clinicians were nurses (28% vs. 19% controls), who seemed to recruit at a higher rate. More than half the children in the intervention arm were recruited by nurses compared with less than a third amongst the controls, although both GPs (median 3 children vs. 1 amongst controls, p=0.13) and nurses (median 12 children vs. 3 amongst controls, p=0.08) in the intervention arm recruited at higher rates compared with the controls.

Demographics

There was no marked difference between arms with regard to gender, ethnicity or deprivation score (Table 2). The median age of the children in the intervention group was younger compared to the

For peer review only - http://bmjopen.bmj.com/site/about/guidelines.xhtml

control group (2 years vs. 3 years, $p=0.03$) and the intervention children lived in households with proportionally more smokers (29% vs. 17% controls, $p=0.002$).

Symptoms, signs and health outcomes

The majority of symptoms and signs were more prevalent amongst the intervention children (Table 3) who had a longer illness duration prior to consultation ($p=0.03$), a higher respiratory rate ($p<0.001$), higher wheeze prevalence ($p=0.007$), higher illness severity scores, measured by both the carer ($p=0.045$) and clinician ($p=0.02$) and children where the clinician had a 'gut feeling' that something was wrong ($p=0.03$). There was little evidence of a difference between days to cough resolution (median 14 days, IQR 9-25 days in intervention arm compared with a median of 13 days, IQR 8-27days in the control arm, $p=0.77$) or follow-up appointments (primary or secondary: 2.1% vs. 1.0% controls, $p=0.33$). There was a slightly higher proportion of children in the intervention arm attending Emergency Departments in the 30 days post recruitment (2.1% vs. 0.5% controls, $p=0.14$) although none of these children received treatment. There was one hospital admission in the 30 days post recruitment; the child was in the intervention arm and was discharged on the same day with viral induced wheeze.

Antibiotic prescribing

In the cohort study upon which the algorithm was based,[19] (conducted between 2011 and 2013) the overall antibiotic prescribing rate at consultation was 37.2%, of which 28.0% was immediate and 9.2% delayed. As Table 4 shows, in the intervention group of the CHICO feasibility trial the overall antibiotic prescribing rate at consultation was 25% (19.9% immediate and 5.1% delayed) – demonstrating a marked reduction in both immediate and delayed prescribing compared with estimates from a few years earlier. However, the prescribing rate amongst the control children in the study was even lower (15.8%, 14.4% immediate and 1.4% delayed).

<u>Fidelity and acceptability of the intervention</u>

None of the data collection web-sessions during consultation were abandoned and no technical issues were reported with the web-based tool after early teething problems (largely due to the use of older web-browsers). According to the available web page time-stamp data, the median time taken to complete the intervention was 5-6 minutes. This was consistent with clinicians reporting in the interviews that the intervention added 5-10 minutes onto their consultations. Some clinicians said they were reassured that hospitalisation risk agreed with their clinical judgement and some felt it was a useful backup for carers. The pages describing the background to the trial were only accessed on 29 occasions, although we are not able to discern whether these were unique visitors, or multiple visits from a few clinicians. Clinicians printed the personalised leaflet for the majority of the carers (92%) but only 4 of the 14 carers interviewed reported receiving and reading the printout.

For peer review only - http://bmjopen.bmj.com/site/about/guidelines.xhtml

For peer review only

Qualitative interviews with clinicians and carers

Interviews were conducted with 24 clinicians (17 general practitioners and 7 nurses) and 14 carers sampled with varying characteristics (Table 5).

Recruitment

Carers perceived the data burden across the period of the study to be light. Clinicians, carers and children all liked the recruitment packs, which had been designed by a graphic designer, to be colourful and child-friendly. Clinicians in both arms reported recruiting less unwell children, for whom consultations were expected to be quicker to treat and therefore easier to combine with additional research activity. Although clinicians commented that the intervention was easy and quick to use compared with other studies, it still added time to the consultation. Practices with an appointment system that channelled eligible children towards active recruiters, who were often minor illness nurses, had high recruitment rates. In contrast, practices that normally channelled same day appointments towards particular (non-recruiting) clinicians tended to be very low recruiters, despite attempts to get eligible patients redirected to recruiters.

Using the Intervention

Some clinicians in both arms initiated recruitment only after they had completed their 'normal consultation'. Most said this was because engaging with the web-based tool interfered with their 'normal consultation', others that they needed to assess the child for serious illness before deciding whether to include them in the trial. Clinicians reported a high degree of awareness of the problem of high antibiotic prescribing rates and described a range of other concurrent initiatives aimed at reducing antibiotic prescribing.

Intervention clinicians rarely reported using the CHICO algorithm results as a decision aid. Their reasons for this included: i) their 'normal consultation' including treatment decision was undertaken before initiating the intervention; ii) they decided early in the consultation that they didn't need to prescribe and therefore extra information was not useful (perhaps exacerbated where they chose not to recruit sicker children); iii) they lacked trust in the algorithm. Clinicians said the questions/information included in the intervention were more detailed than they usually asked, but none felt it had influenced or changed their decision. They were reassured when the reported risk of hospitalisation agreed with their clinical judgement, and could be used as a useful backup to present to carers. If the hospitalisation risk or advice provided was at odds with their clinical judgement a few clinicians said that they ignored the advice.

Most carers said the CHICO consultation appeared as normal to them, while two said the history taking or explanations were more detailed. Few carers were aware of the reported risk of

For peer review only - http://bmjopen.bmj.com/site/about/guidelines.xhtml

hospitalisation or subsequent advice from the CHICO intervention; one was reassured by the low risk advice. Two had experiences of the clinician prescribing antibiotics despite CHICO advice.

The carer leaflet

Clinicians valued having the leaflet to print out for carers highly; some said it was a useful alternative to a prescription. Some felt it backed up their recommendations and others felt it was good for carers to have printed information. Carers had mixed views about the leaflet; some felt it didn't tell them anything they didn't know already; others said it gave them some useful information; and in one case the carer reported that it prevented a re-consultation.

Health Economics

The review of patient practice records and the online data collection system, supported by telephone calls to carers, provided a comprehensive source of information on NHS resource use, although data missingness increased with symptom duration. Almost 99% of carers reported some resource use. The initial consultation was estimated to take 15.29 minutes in the intervention arm, compared with 10 minutes in the control arm. Cost data were positively skewed, reflecting limited NHS costs other than for the initial GP consultation.

Mean NHS costs per patient from available cases (n=494) were £54.62 in control practices, and £78.78 in intervention practices. The difference of £24.15 is associated with a bias corrected and accelerated 95% confidence interval of £18.57 to £32.98. Mean per-patient costs were primarily determined by the costs of the initial consultation, which constitute 73% of NHS costs in the control arm and 78% in the intervention arm. Personal and productivity costs were modest.

Only one in four recruited children were in the age range of 5-11 suitable for the CHU-9D instrument; moreover, only 44 (9% of all participants) provided baseline and follow-up data from the week of resolution to facilitate the estimation of quality adjusted life years (QALYs). The data on these children tentatively suggest that their quality of life returned to population norms after the baseline appointment.

For peer review only - http://bmjopen.bmj.com/site/about/guidelines.xhtml

Discussion

The feasibility study showed high recruitment and retention. Quantitative and qualitative data confirm the intervention was acceptable to the clinicians and widely used, but post-randomisation differential recruitment undermined the feasibility trial's ability to determine unbiased estimates of intervention effect.

Differential recruitment was quite marked in terms of who recruited, the number recruited, the background characteristics of the children and their signs and symptoms. Many of the symptoms and signs were more prevalent among the intervention children. In the qualitative interviews, clinicians from both arms of the trial reported preferential recruitment of less unwell children as these were quicker to deal with and easier to combine with the research. This was particularly true in the control arm, where larger numbers of well children, relative to the intervention arm, were recruited. A future trial design needs to minimise or avoid this post-randomisation recruitment bias.

A Hawthorne effect (whereby clinicians modified their behaviour in response to their awareness of being in the trial) may also have been acting to reduce prescribing in both arms. Although we did not overtly advertise that the trial was focussing on reducing antibiotic prescribing, the clinicians may have inferred the aims of the trial. Our previous work with these practices may have alerted some clinicians to our aims, coupled with national and local campaigns to increase awareness of antimicrobial resistance. The control clinicians used the same web-based tool during consultation to record study data. Thus our data collection in itself amongst the controls may have inadvertently increased risk perception (by providing risk information, albeit partially obscured among the data collected that was previously unavailable to clinicians) as well as provide 'test' conditions. We also restricted the maximum recruitment per practice to 30 children which reduced run-in time for clinician behaviour to change from initial feelings of 'test-conditions' to 'usual practice'. However, these Hawthorn effects do not explain the lower prescribing rates in the control arm.

The web-based data collection tool and review of medical notes were appropriate vehicles for resource-use data collection. The principal driver of higher costs in the intervention group were longer initial GP appointments, when the web-based system was being used. There was little evidence on quality of life because of limited data availability.

<u>Strengths and weaknesses</u>

There are many strengths to this feasibility trial. Recruitment was so rapid that we stopped a month earlier than planned, with robust data collection and few missing values. We adequately resourced the trial to achieve excellent follow-up with little attrition, building up relationships with the families and

For peer review only - http://bmjopen.bmj.com/site/about/guidelines.xhtml

using several types of communication media as an effective way of obtaining data for our web-based entry system. We reduced variation in practice recruitment levels, compared to our previous cohort study, with a higher median number of patients per practice and fewer non-recruiting practices. None of the intervention web-sessions were abandoned and clinicians thought it was quick and easy to use compared to other interventions. Clinicians also highly valued having the personalised leaflet as an alternative to a prescription.

Apart from differential recruitment and the potential of inducing a Hawthorne effect, there were other weaknesses in this trial that we can learn from. The intervention was designed to be used as part of a decision aid, but it became apparent that some clinicians only used the intervention after the treatment decision was made. Using the intervention added around five minutes to consultation time, which could be reduced in future. Some of this time was to record research data and consideration needs to be given as to whether these data can be collected outside the consultation either from the medical notes or at practice level. The intervention was a stand-alone system and might be more acceptable and easier to use if embedded within the existing practice records system. Although, as previously stated, clinicians liked the personalised leaflets, carers were less enthusiastic. Further development through parent consultation would be recommended for a future trial.

Results in context with other research

We have constructed a complex intervention associated with lower antibiotic prescribing rates (from 37% to 25%) compared with data from similar practices in the same area a couple of years earlier[19], but an even lower rate among concurrent controls (16%). Local data over a four-year period suggest the extremely low prescribing rates among the control practices do not reflect usual practice, which may be due to differential recruitment.

Antibiotic prescription data from a large primary care database, covering 537 UK general practices during 1995–2011 suggest the rate of prescribing for coughs and colds initially fell around the millennium but by 2011 had risen to 40%.[27] Our estimate of 37% amongst children from a large cohort study conducted between 2011 and 2013[19] is a robust estimate and a reduction in prescribing by almost a third to 25% is worth pursuing. To do this, a future trial must overcome the dual problems of differential recruitment and the Hawthorne effect.

A primary care feasibility trial in back pain[28] that experienced differential recruitment recommended identifying patients beforehand or for independent researchers to do the recruitment. Given the acute nature of childhood cough it would not be possible to identify the children beforehand and the logistics and cost of using independent researchers at different primary care sites would be prohibitive. One approach would be to select primary care sites that adopt a triage system for children

For peer review only - http://bmjopen.bmj.com/site/about/guidelines.xhtml

with cough and work more closely with these different systems to help reduce selection bias. Alternatively a more 'light touch' design could be adopted at the practice level using routinely collected practice-wide data and practice level consent, negating the need to consent and recruit individual patients post randomisation. The unit of interest would be the practice rather than the patient so all patients seen in a specified time would be included. Amoxicillin suspension is the main antibiotic given to children with acute cough and RTI and has been used previously [29] as a proxy marker to assess antibiotic prescribing at the practice level. This would also remove the possibility of individual consent, so considerations of research ethics would be needed.

The Hawthorne effect is not uncommon in clinical trials in general or indeed in trials specifically aimed at reducing antibiotic prescribing[30], but the mechanisms of effect and magnitude are not well understood.[31] Given government campaigns targeting antimicrobial resistance and a need to reduce antibiotic prescribing it would be difficult to avoid such an effect completely.

Conclusions

Many valuable lessons have been learnt from this feasibility study, and a redesign is required for any future trial. To negate differential recruitment and reduce the possibility of a Hawthorne effect a 'light touch' efficient design is needed that avoids patient recruitment at the clinician level and utilises data already routinely collected by the practices themselves. Better training in the use of the intervention and encouragement to use the tool as part of the consultation process would be facilitated if the intervention is embedded within current practice systems. Removing the need for clinicians to recruit patients or enter data other than that required for the tool would both reduce the time added to consultations and preserve usual practice amongst the control clinicians.

For peer review only

Competing interests

Financial competing interests. The authors declare that they have no competing interests. Non-financial competing interests. The authors declare that they have no competing interests.

Authors' contributions

ADH, PJL, PSB, NMR, JH, SH, NF, TJP and JI were responsible for developing the research question. PSB, ADH, JI, PJL, NMR, ST, SH, JH, CC, NF, AG, CJ and TJP were responsible for the study design and collection of data. ST, PSB and TJP were responsible for quantitative analysis, CC and JH were responsible for qualitative analysis, PD and SH were responsible for the economic evaluation. NMR and ST were responsible for study management, monitoring and co-ordination. PB and ST drafted the paper. All authors read, commented on and approved the final manuscript.

UK clinical research network (UKCRN) portfolio registration

The cohort study is registered on UK Clinical Research Network Portfolio as 'The CHICO (Children's Cough) Feasibility Trial' reference number 16891.

Funding statement

The TARGET Programme is funded by the National Institute for Health Research's Programme Grant for Applied Research Programme. This paper presents independent research funded by the National Institute for Health Research (NIHR) under its Programme Grant for Applied Research (Grant Reference Number RP-PG-0608-10018). The views expressed are those of the author(s) and not necessarily those of the NHS, the NIHR or the Department of Health.

NMR's time is currently supported by the National Institute for Health Research (NIHR) Collaboration for Leadership in Applied Health Research and Care West (CLAHRC West) at University Hospitals Bristol NHS Foundation Trust.

Data sharing statement

The full data set will be made available when all studies described within the protocol are complete and published. Application for the data to be released should be made in writing to Dr Peter Blair (PI for this feasibility study) via the Freedom of Information Officer at the University of Bristol

Acknowledgements

The authors are extremely grateful to the children, carers and families who have participated in the study, all GP practices including recruiting clinicians, administrative and research contacts and all other staff whose participation made this study possible. We would like to thank our parent and

For peer review only - http://bmjopen.bmj.com/site/about/guidelines.xhtml

clinician advisory groups for their time and thoughts (PAG members: Hannah Walter, Kelly McCauley, Tasha Buckland-Smith, Sandra Watt, Kathryn Johnson, Lucy Ashton, Louise Winter, Lee Williams, Liz Williams. GAG members: Elaine Luntz, Kirsty Alexander, Joanna Williams, Mike Rossdale, Knut Schroeder, Lucy Jenkins, Terry Kemple, Marion Steiner, Alex Hickson, Chris Milsom, Gina Wills, and Jo Jenkins). We thank colleagues from the TARGET Programme, the TARGET Programme Management Group and the TARGET Programme Steering Committee (Sandra Eldridge, Nick Francis, Paul Little, Joe Kai, Victoria Senior, Anna Thursby-Pelham and Jon Hayhurst) for their time, expertise and support. We are grateful to the following individuals who have helped with the study; Judy Cordell, Elizabeth Derodra, Sandra Mulligan, Sara Tonge and Hanna Oakes. Thanks goes to Kinetic Creative Limited for developing our web-based intervention in collaboration with the CHICO team. We are grateful for the support of the West of England Primary Care Network and the UK Clinical Research Networks involved in the study, specifically the West of England Clinical Research Network and the Business and Finance Officer Julie Brooks. The randomisation process for this trial was designed and delivered in collaboration with Daisy Gaunt and Chris Metcalfe from the Bristol Randomised Trials Collaboration (BRTC), a UKCRC Registered Clinical Trials Unit in receipt of National Institute for Health Research CTU support funding.

For peer review only

References

1) Hay AD, Heron J, Ness A, the ALSPAC study team. The prevalence of symptoms and consultations in pre-school children in the Avon Longitudinal Study of Parents and Children (ALSPAC): a prospective cohort study. *Fam Pract* 2005; 22(4):367-374.

2) Hollinghurst S, Gorst C, Fahey T, Hay A. Measuring the financial burden of acute cough in pre-school children: a cost of illness study. *BMC Family Practice* 2008; 9(1):10.

3)Lucas P, Cabral C, Hay A,el al. A systematic review of parent and clinician views and perceptions that influence prescribing decisions in relation to acute childhood infections in primary care. *Scandinavian Journal of Primary Health Care* 2015, **0**:1-10.

4) National Institute for Clinical Excellence (NICE): Department of Health. Respiratory tract infections: prescribing of antibiotics for self-limiting respiratory tract infections in adults and children in primary care. CG69. London: *National Institute for Clinical Excellence* 2008.

5) Department of Health. UK Five Year Antimicrobial Resistance Strategy 2013 to 2018. In: *Health* Do, ed., 2013.

6) Thompson PL, Spyridis N, Sharland M, et al. Changes in clinical indications for community antibiotic prescribing for children in the UK from 1996-2006: will the new NICE prescribing guidance on upper respiratory tract infections be ignored? *Arch Dis Child* 2009; **94**(5):337-340

7) World Health Organisation, Antimicrobial resistance: Global Report on Surveillance 2014, *World Health Organisation: France*. p. 256.

8) O'Neill, J., Tackling drug-resistant infections globally: final report and recommendations, in *Review on Antimicrobial Resistance*. 2016.

9) *World Economic Forum, Global Risks* 2014. 2014. p. 60

10) Vodicka TA, Thompson M, Lucas P, Heneghan C, Blair PS, Buckley DI, Redmond N, Hay AD; TARGET Programme team. Reducing antibiotic prescribing for children with respiratory tract infections in primary care: a systematic review. *Br J Gen Pract*. 2013 Jul; 63(612):e445-54.

11) Andrews T, Thompson M, Buckley DI, Heneghan C, Deyo R, Redmond N, Lucas PJ, Blair PS, Hay AD. Interventions to influence consulting and antibiotic use for acute respiratory tract infections in children: a systematic review and meta-analysis. *PLoS One.* 2012; 7(1):e30334.

12) Cabral, C. Ingram, J. Hay, A. and Horwood, J. "They just say everything's a virus" - parent's judgment of the credibility of clinician communication in primary care consultations for respiratory tract infections in children: A qualitative study. *Patient Education and Counseling* 2014; 95 (2), 248–253.

13) Ingram J, Cabral C, Hay AD, Lucas PJ, Horwood J. Parents' information needs, self-efficacy and influences on consulting for childhood respiratory tract infections: a qualitative study. *BMC Family Practice* 2013; 14:106

For peer review only - http://bmjopen.bmj.com/site/about/guidelines.xhtml

14) Cabral, CL, Horwood, JP, Hay, AD & Lucas, PJ. How communication affects prescription decisions in consultations for acute illness in children: a systematic review and meta-ethnography. *BMC Family Practice* 2014; 15:63.

15) Cabral, C. Ingram, JC, Lucas, PJ, Redmond, NM, Kai, J, Hay, AD & Horwood, JP. 'The influence of clinical communication on parents' antibiotic expectations for children with Respiratory Tract Infections'. *Annals of Family Medicine* 2016; 14(2): 141-147.

16) Cabral, C. Lucas, P.L. Ingram, J.C. Hay, A. Horwood, J. It's safer to... Safety for parents consulting and clinicians prescribing antibiotics for children with respiratory tract infections: an analysis across four linked qualitative studies. Social Science & Medicine 2015; 136-137:156- 164.

17) Horwood, J. Cabral, C. Hay, A. Ingram, J. Primary healthcare practitioners' diagnostic and antibiotic prescribing decisions in children's consultations for respiratory tract infections. British Journal of General practice Mar 2016; 66 (644): e207 -e213.

18) Redmond NM, Davies R, Christensen H, Blair PS et al (11). The TARGET cohort study protocol: a prospective primary care cohort study to derive and validate a clinical prediction rule to improve the targeting of antibiotics in children with respiratory tract illnesses. *BMC Health Serv Res*. 2013 Aug 17;13(1):322. doi: 10.1186/1472-6963-13-322.

19) Hay AD, Redmond NM, Turnbull S, Christensen H, Thornton, Little P, Thompson M, Delaney B, Lovering AM, Muir P, Leeming JP, Vipond B, Stuart B, Peters TJ, Blair PS. Development and internal validation of a clinical rule to improve antibiotic use in children presenting to primary with acute respiratory tract infection and cough: the 'TARGET' prognostic cohort study. *Lancet Respiratory Medicine* 2016 [in press].

20) Turnbull SL,, Redmond NM, Lucas P, Cabral C, Ingram J, Hollinghurst S, Hay AD, Peters, TJ, Horwood J, Little P, Francis N & Blair PS. The CHICO (Children's Cough) Trial protocol: a feasibility randomised controlled trial investigating the clinical and cost-effectiveness of a complex intervention to improve the management of children presenting to primary care with acute respiratory tract infection. *BMJ Open, 2015,* 5**,** e008615.

21) Chan AW, Tetzlaff JM,Gøtzsche PC, et al. SPIRIT 2013 explanation and elaboration: guidance for protocols of clinical trials.*BMJ* 2013; **346**: e7586

22) Campbell MK, Piaggio G, Elbourne DR, Altman DG; for the CONSORT Group. Consort 2010 statement: extension to cluster randomised trials. BMJ. 2012 Sep 4;345:e5661. PMID: 22951546

23) Curtis L. Unit Costs of Health and Social Care 2013. Canterbury: Personal Social Services Research Unit; 2013.

24) Department of Health. Reference Costs 2012/13. London 2013.

25) Braun V, Clarke V. Using thematic analysis in psychology. *Qualitative Research in Psychology*. 2006;3(2):77-101.

For peer review only - http://bmjopen.bmj.com/site/about/guidelines.xhtml

26) Meyrick J. What is good qualitative research? A first step towards a comprehensive approach to judging rigour/quality. *J Health Psychol*. 2006;11(5):799-808

27) Hawker JI, Smith S, Smith GE, Morbey R, Johnson AP, Fleming DM, Shallcross L, Hayward AC. Trends in antibiotic prescribing in primary care for clinical syndromes subject to national recommendations to reduce antibiotic resistance, UK 1995-2011: analysis of a large database of primary care consultations. *J Antimicrob Chemother*. **2014** Dec;69(12):3423-30. doi: 10.1093/jac/dku291. Epub

28) Farrin A, Russell I, Torgerson D, Underwood M & Team UBT. Differential recruitment in a cluster randomized trial in primary care: the experience of the UK back pain, exercise, active management and manipulation (UK BEAM) feasibility study. *Clin Trials* 2005, 2, 119-24.

29) Butler CC, Simpson SA, Dunstan F et al. Effectiveness of multifaceted educational programme to reduce antibiotic dispensing in primary care: practice based randomised controlled trial. *BMJ* 2012, 344.

30) Mangion-Smith R, Elliott MN, McDonald L & McGlynn EA. An observational study of antibiotic prescribing behavior and the Hawthorne effect. Health Services Research 2002, 37, 1603-23.

31) McCambridge J, Witton J, Elbourne DR. Systematic review of the Hawthorne effect: new concepts are needed to study research participation effects. *J Clin Epidemiol* 2014, 67, 267-77.

review only

For peer review only - http://bmjopen.bmj.com/site/about/guidelines.xhtml

Table 1: Comparison of the clinician variables in the two arms of the trial

	Control		Intervention		
	n/N or Median	% or (IQR)	n/N or Median	% or (IQR)	p-value
**Clinician profile**					
Number of GCP trained clinicians	34/58	58.6%	32/46	69.6%	0.25[1]
Years since qualified	22	(14-27)	23	(17-27)	0.38[2]
**Clinicians who recruited**					
Number of recruiting clinicians	30/58	51.7%	34/46	73.9%	0.021[1]
Number of recruiting nurses	7/11	63.6%	11/13	84.6%	0.36[3]
Number of recruiting GPs	23/47	48.9%	23/33	69.7%	0.064[1]
**Number of children recruited by clinician type**					
Number of children recruited by nurses	65/209	31.1%	155/292	53.1%	<0.001[1]
Number of children recruited by GPs	144/209	68.9%	137/292	46.9%	<0.001[1]
**Recruitment of children**					
Median number of children recruited per clinician	1	(0-4)	3	(0-11)	0.014[2]
Median number recruited per nurse	3	(0-7)	12	(3-17)	0.13[2]
Median number recruited per GP	0	(0-4)	2	(0-4)	0.077[2]

1 Chi-square test
2 Mann Whitney test
3 Fisher's Exact test

Table 2: Demographic Profile in each arm of the trial

	Control		Intervention		p-value
	Median	IQR	Median	IQR	
Child age	3 years	1-5 years	2 years	1-4 years	0.028[1]
Home IMD* score	12.1	6.9-21.5	12.1	6.5-23.9	0.74[1]
	n/N	%	n/N	%	
Gender (male)	105/209	50.2%	139/292	47.6%	0.56[2]
Any smoker in household	34/206	16.5%	82/288	28.5%	0.020[2]
**Ethnicity:**					
White	187/209	89.5%	251/292	86.0%	
Mixed	8/209	3.8%	17/292	5.8%	0.72 (4d.f.)[2]
Asian or Asian British	7/209	3.4%	9/292	3.1%	
Black or Black British	5/209	2.4%	11/292	3.8%	
Other ethnic group	2/209	1.0%	4/292	1.4%	

* Index of multiple deprivation
1 Mann Whitney test
2 Chi-square test

For peer review only - http://bmjopen.bmj.com/site/about/guidelines.xhtml

Table 3: Comparison of children's symptoms and signs

	Control		Intervention		
	**Median**	**(IQR)**	**Median**	**(IQR)**	**p-value**
**Symptom or sign**					
Illness duration prior to consultation	5 days	(3-13)	7 days	(4-14)	0.034[1]
Clinician reported illness severity score (0-10)	3	(2-4)	3	(2-4)	0.022[1]
	**Mean**	**SD**	**Mean**	**SD**	
Carer reported illness severity score (0-10)	4.89	1.75	5.23	1.94	0.045[2]
	**n/N**	**%**	**n/N**	**%**	
Severe cough (24 hrs prior to consultation)	41/208	19.7%	79/292	27.1%	0.058
Severe fever (24 hrs prior to consultation)	15/208	7.2%	26/292	8.9%	0.50
Moderate or severe vomiting (24 hours prior)	18/208	8.7%	41/292	14.0%	0.066
High temperature (≥37.8°C)	29/208	13.9%	29/292	9.9%	0.17
High respiratory rate*	11/208	5.3%	44/292	15.1%	<0.001
Inter subcostal recession	5/208	2.4%	11/292	3.8%	0.39
Wheeze	17/208	8.2%	48/292	16.4%	0.007
Crackles or Crepitations	37/208	17.8%	65/292	22.3%	0.22
Clinician had 'gut feeling' something was wrong	12/208	5.8%	34/292	11.6%	0.025

* Using age-related cut-offs
1 Mann Whitney test
2 T-Test
3 Chi-square test

Table 4: Antibiotics prescribed at consultation in the two arms of the trial

**Prescribing**	**Control**		**Intervention**		**p-value**
	N	%	N	%	
No antibiotics prescribed	176/209	84.3	219/292	75.0	0.018 (2 d.f.)
Immediate antibiotics prescribed	30/209	14.4	58/292	19.9	
Delayed antibiotics prescribed	3/209	1.4	15/292	5.1	

For peer review only - http://bmjopen.bmj.com/site/about/guidelines.xhtml

Table 5: Description of clinicians & carers sampled for the qualitative analysis

**Clinician sample**	**Characteristic**	N	**%**
Total clinicians in sample		24	100%
Study Arm	*Intervention*<br>*Control*	12<br>12	50%<br>50%
Clinician type	*GP*<br>*Nurse*	17<br>7	70.8%<br>29.2%
Clinician recruitment rate	*> 20 children*<br>*10-20 children*<br>*< 10 children*	4<br>9<br>11	16.7%<br>37.5%<br>45.8%
Clinician antibiotic Prescribing rate	>25%<br>15-24%<br><15%	5<br>9<br>10	21%<br>38%<br>42%
**Carer sample**	**Characteristic**	N	**%**
Total carers in sample		14	100%
IMD quintile*	*1 (most deprived)*<br>*2*<br>*3*<br>*4*<br>*5 (most affluent)*	1<br>5<br>2<br>3<br>3	7.1%<br>35.7%<br>14.3%<br>21.4%<br>21.4%
Child age (in years)	*<2*<br>*2-4*<br>*5+*	8<br>4<br>2	57.1%<br>28.6%<br>14.3%
Hospitalisation Risk and Treatment decision	*Low Risk – Home care*<br>*Low Risk – Delayed antibiotics*<br>*Low Risk – Immediate antibiotics*<br>*Medium Risk - Home care*<br>*Medium Risk - Delayed antibiotics*<br>*Medium Risk – Immediate antibiotics*	5<br>3<br>1<br>3<br>0<br>2	35.7%<br>21.4%<br>7.1%<br>21.4%<br>0.0%<br>14.3%

* Index of Multiple Deprivation based on the postcode of the family home

For peer review only

Table 1: CONSORT 2010 checklist of information to include when reporting a cluster randomised trial

Section/Topic	Item No	Standard Checklist item	Extension for cluster designs	Page No *
**Title and abstract**				
	1a	Identification as a randomised trial in the title	Identification as a cluster randomised trial in the title	Yes (page 1)
	1b	Structured summary of trial design, methods, results, and conclusions (for specific guidance see CONSORT for abstracts)[1,2]	See table 2	Yes (page 2)
**Introduction**				
**Background and objectives**	2a	Scientific background and explanation of rationale	Rationale for using a cluster design	Yes (page 4). For this type of knowledge intervention trial clustering is self evident so explanation not provided (given the word limit)
	2b	Specific objectives or hypotheses	Whether objectives pertain to the the cluster level, the individual participant level or both	Yes (page 4) although this is a feasibility study so the main objectives surround issues of recruitment and acceptability of the intervention
**Methods**				
**Trial design**	3a	Description of trial design (such as parallel, factorial) including allocation ratio	Definition of cluster and description of how the design features apply to the clusters	Yes (page 5)
	3b	Important changes to methods after trial commencement (such as eligibility criteria), with reasons		No important changes made
**Participants**	4a	Eligibility criteria for	Eligibility criteria for clusters	Yes (page 5)

For peer review only

		participants		
	4b	Settings and locations where the data were collected		Yes (page 5)
**Interventions**	5	The interventions for each group with sufficient details to allow replication, including how and when they were actually administered	Whether interventions pertain to the cluster level, the individual participant level or both	Yes (page 5)
**Outcomes**	6a	Completely defined pre-specified primary and secondary outcome measures, including how and when they were assessed	Whether outcome measures pertain to the cluster level, the individual participant level or both	Yes (pages 6 and 7) although these refer specifically to the feasibility study
	6b	Any changes to trial outcomes after the trial commenced, with reasons		No changes made
**Sample size**	7a	How sample size was determined	Method of calculation, number of clusters(s) (and whether equal or unequal cluster sizes are assumed), cluster size, a coefficient of intracluster correlation (ICC or *k*), and an indication of its uncertainty	This was a feasibility trial so no formal sample size required although our pragmatic approach has been described (page 5)
	7b	When applicable, explanation of any interim analyses and stopping guidelines		Not applicable
**Randomisation:**				
**Sequence generation**	8a	Method used to generate the random allocation sequence		Yes (page 5)
	8b	Type of randomisation; details of any restriction (such as blocking and block size)	Details of stratification or matching if used	Yes (page 5)
**Allocation concealment mechanism**	9	Mechanism used to implement the random allocation sequence (such as sequentially numbered containers), describing any	Specification that allocation was based on clusters rather than individuals and whether allocation concealment (if any) was at the cluster level, the individual	Yes (page 5) Simple randomisation used at cluster level, a one-off

For peer review only

		steps taken to conceal the sequence until interventions were assigned	participant level or both	procedure
**Implementation**	10	Who generated the random allocation sequence, who enrolled participants, and who assigned participants to interventions	Replace by 10a, 10b and 10c	
	10a		Who generated the random allocation sequence, who enrolled clusters, and who assigned clusters to interventions	Yes (page 5)
	10b		Mechanism by which individual participants were included in clusters for the purposes of the trial (such as complete enumeration, random sampling)	Yes (page 5)
	10c		From whom consent was sought (representatives of the cluster, or individual cluster members, or both), and whether consent was sought before or after randomisation	Yes (page 5)
**Blinding**	11a	If done, who was blinded after assignment to interventions (for example, participants, care providers, those assessing outcomes) and how		Not applicable (blinding could not be used)
	11b	If relevant, description of the similarity of interventions		Not applicable
**Statistical methods**	12a	Statistical methods used to compare groups for primary and secondary outcomes	How clustering was taken into account	Yes (page 7)
	12b	Methods for additional analyses, such as subgroup analyses and adjusted analyses		Yes (page 7)
**Results**				

For peer review only

**Participant flow (a diagram is strongly recommended)**	13a	For each group, the numbers of participants who were randomly assigned, received intended treatment, and were analysed for the primary outcome	For each group, the numbers of clusters that were randomly assigned, received intended treatment, and were analysed for the primary outcome	Yes (page 8)
	13b	For each group, losses and exclusions after randomisation, together with reasons	For each group, losses and exclusions for both clusters and individual cluster members	Yes (page 8)
**Recruitment**	14a	Dates defining the periods of recruitment and follow-up		Yes (page 8)
	14b	Why the trial ended or was stopped		Not applicable
**Baseline data**	15	A table showing baseline demographic and clinical characteristics for each group	Baseline characteristics for the individual and cluster levels as applicable for each group	Yes (page 20)
**Numbers analysed**	16	For each group, number of participants (denominator) included in each analysis and whether the analysis was by original assigned groups	For each group, number of clusters included in each analysis	Yes (page 8 and page 20)
**Outcomes and estimation**	17a	For each primary and secondary outcome, results for each group, and the estimated effect size and its precision (such as 95% confidence interval)	Results at the individual or cluster level as applicable and a coefficient of intracluster correlation (ICC or k) for each primary outcome	Yes (page 21) but only to a point as this is a feasibility trial
	17b	For binary outcomes, presentation of both absolute and relative effect sizes is recommended		Yes (page 21)
**Ancillary analyses**	18	Results of any other analyses performed, including subgroup analyses and adjusted analyses, distinguishing pre-specified from exploratory		Not applicable, the main analysis surrounds recruitment issues and qualitative work as part of a feasibility study
**Harms**	19	All important harms or unintended effects in each		No adverse events to report

		group (for specific guidance see CONSORT for harms[3])		
**Discussion**				
**Limitations**	20	Trial limitations, addressing sources of potential bias, imprecision, and, if relevant, multiplicity of analyses		Yes (pages 12 and 13) and summarised as requested (page 3)
**Generalisability**	21	Generalisability (external validity, applicability) of the trial findings	Generalisability to clusters and/or individual participants (as relevant)	Yes (pages 13 and 14)
**Interpretation**	22	Interpretation consistent with results, balancing benefits and harms, and considering other relevant evidence		Yes (pages 13 and 14)
**Other information**				
**Registration**	23	Registration number and name of trial registry		Yes (page 15)
**Protocol**	24	Where the full trial protocol can be accessed, if available		The trial protocol paper is referenced (page 5)
**Funding**	25	Sources of funding and other support (such as supply of drugs), role of funders		Yes (page 15)

** Note: page numbers optional depending on journal requirements*

For peer review only

[1] Hopewell S, Clarke M, Moher D, Wager E, Middleton P, Altman DG, et al. CONSORT for reporting randomised trials in journal and conference abstracts. *Lancet* 2008, 371:281-283

[2] Hopewell S, Clarke M, Moher D, Wager E, Middleton P, Altman DG at al (2008) CONSORT for reporting randomized controlled trials in journal and conference abstracts: explanation and elaboration. *PLoS Med* 5(1): e20

[3] Ioannidis JP, Evans SJ, Gotzsche PC, O'Neill RT, Altman DG, Schulz K, Moher D. Better reporting of harms in randomized trials: an extension of the CONSORT statement. *Ann Intern Med* 2004; 141(10):781-788.

For peer review only

BMJ Open

Feasibility cluster randomised controlled trial of a within-consultation intervention to reduce antibiotic prescribing for children presenting to primary care with acute respiratory tract infection and cough

Journal:	*BMJ Open*
Manuscript ID	bmjopen-2016-014506.R1
Article Type:	Research
Date Submitted by the Author:	02-Feb-2017

Complete List of Authors:	Blair, Peter; University of Bristol, School Of Social & Community Medicine<br>Turnbull, Sophie; University of Bristol, School of Social and Community Medicine<br>Ingram, Jenny; Bristol University , School of Social & Community Medicine<br>Redmond, Niamh; University of Bristol, Academic Unit of Primary Health Care<br>Lucas, Patricia; Unversity of Bristol, School for Policy Studies<br>Cabral, Christie; University of Bristol, School of Social and Community Medicine<br>Hollinghurst, Sandra; University of Bristol, Community Based Medicine<br>Dixon, Padraig; University of Bristol, School of Social and Community Medicine, University of Bristol; School of Social and Community Medicine<br>Peters, Tim; University of Bristol, Department of Community Based Medicine<br>Horwood, Jeremy; University of Bristol, UK, School of Social and Community Medicine<br>Little, Paul; University of Southampton, Primary Care and Population Science;<br>Francis, Nick A.; Cardiff University School of Medicine, School of Medicine<br>Gilbertson, Anna; University of Bristol, School Of Social & Community Medicine<br>Jameson, Catherine; University of Bristol, School Of Social & Community Medicine<br>Hay, Alastair; University of Bristol, Academic Unit of Primary Health Care

**Primary Subject Heading**:	General practice / Family practice
Secondary Subject Heading:	Respiratory medicine, Infectious diseases
Keywords:	Cough, Antibiotics, Respiratory Tract Infections (RTIs), PRIMARY CARE, Children, Feasibility

SCHOLARONE™
Manuscripts

For peer review only - http://bmjopen.bmj.com/site/about/guidelines.xhtml

For peer review only

Feasibility cluster randomised controlled trial of a within-consultation intervention to reduce antibiotic prescribing for children presenting to primary care with acute respiratory tract infection and cough

Blair PS[1], Turnbull S[2], Ingram JC[1], Redmond NM[2,3], Lucas PJ[4], Cabral C[2], Hollinghurst S[2,3], Dixon P[5], Peters TJ[3,6], Horwood J[2,3], Little P[7], Francis N[8], Gilbertson A[2], Jameson C[2], Hay AD[2].

[1] Centre for Child and Adolescent Health, School of Social and Community Medicine, University of Bristol UK.

[2] Centre for Academic Primary Care, School of Social and Community Medicine, University of Bristol, UK

[3] National Institute for Health Research Collaborations for Leadership in Applied Health Research and Care West (NIHR CLAHRC West), University Hospitals Bristol NHS Foundation Trust, UK

[4] School for Policy Studies, University of Bristol, UK

[5] School of Social and Community Medicine, University of Bristol, UK

[6] School of Clinical Sciences, University of Bristol, UK

[7] Department of Primary Care & Population Sciences, University of Southampton, UK

[8] Division of Population Medicine, Cardiff University, UK

Corresponding author

Dr Peter S Blair, Reader in Medical Statistics
Level D, St Michael's Hospital, Southwell St, Bristol, BS2 8EG.
Phone: 01173425145
Fax: 01173425154
Email: p.s.blair@bris.ac.uk

Trial registration: ISRCTN23547970

UKCRN study ID: 16891

Keywords: Randomised controlled trial (RCT), Feasibility, Children, Respiratory tract infections (RTIs), Primary care, Cough, Antibiotics (or Anti-microbial agents)

Main paper Word count: 4313 words

For peer review only

For peer review only - http://bmjopen.bmj.com/site/about/guidelines.xhtml

Abstract

Objective: To investigate recruitment and retention, data collection methods and the acceptability of a 'within-consultation' complex intervention designed to reduce antibiotic prescribing.

Design: Primary care feasibility cluster randomised controlled trial.

Setting: 32 general practices in South West England recruiting children from October 2014 to April 2015.

Participants: Children (aged 3 months to <12 years) with acute cough and respiratory tract infection (RTI).

Intervention: A web-based clinician-focussed clinical rule to predict risk of future hospitalisation and a printed leaflet with individualised child health information for carers, safety-netting advice and a treatment decision record.

Controls: Usual practice, with clinicians recording data on symptoms, signs, and treatment decisions.

Results: Of 542 children invited, 501 (92.4%) consented to participate, a month ahead of schedule. Antibiotic prescribing data were collected for all children, follow-up data for 495 (98.8%) and NHS resource use data for 494 (98.6%). The overall antibiotic prescribing rates for children's RTIs were 25% and 15.8% (p=0.018) in intervention and control groups respectively. We found evidence of post-randomisation differential recruitment: the number of children recruited to the intervention arm was higher (292 vs. 209); over half were recruited by prescribing nurses compared with less than a third in the control arm; children in the intervention arm were younger (median age 2 vs. 3 years controls, p=0.03) and appeared to be more unwell than those in the control arm with higher respiratory rates (p<0.0001), wheeze prevalence (p=0.007) and global illness severity scores assessed by carers (p=0.045) and clinicians (p=0.01). Interviews with clinicians confirmed preferential recruitment of less unwell children to the trial, more so in the control arm.

Conclusion: Differential recruitment may explain the paradoxical antibiotic prescribing rates. Future cluster level studies should consider designs which remove the need for individual consent post-randomisation and embed the intervention within electronic primary care records.

Trial registration number: ISRCTN 23547970

Abstract Word count: 298/300 words

For peer review only - http://bmjopen.bmj.com/site/about/guidelines.xhtml

Strengths & Limitations

- Recruitment was successful with robust data collection and few missing values.
- We adequately resourced the trial, achieving excellent follow-up with little attrition
- We reduced variation in practice recruitment levels, compared to our previous cohort study.
- None of the intervention web-sessions were abandoned and clinicians thought it was quick and easy to use compared to other interventions.
- Clinicians valued the personalised leaflet as an alternative to a prescription although carers were less enthusiastic.
- The intervention was designed to be used as an antibiotic treatment decision aid, but it became apparent that some clinicians only used the intervention after deciding treatment.
- Using the intervention added around five minutes to consultation time. Some of this time was to record research data and consideration needs to be given as to whether these data can be collected outside the consultation.
- The intervention was a stand-alone system and might be more acceptable and easier to use if embedded within the existing practice records system.
- Differential recruitment was quite marked, in particular the intervention children were significantly more unwell. A future trial design needs to minimise or avoid this post-randomisation recruitment bias.
- The control clinicians used the same web-based data collection system during consultation to record study data. Thus our data collection in the controls may have inadvertently increased risk perception as well as providing the ‘test’ conditions to encourage a Hawthorne effect.

For peer review only

For peer review only - http://bmjopen.bmj.com/site/about/guidelines.xhtml

Introduction

Respiratory tract infections (RTIs) in children are extremely common and costly to service providers, families and employers in the UK.[1,2] Clinicians often prescribe antibiotics "just in case", if they feel uncertain about patient health or legal outcomes.[3] Clinical uncertainty in primary care regarding the diagnosis and best management of RTIs has led to the inappropriate use of existing antibiotics, which, combined with the slowing in development of new antibiotics, is associated with antimicrobial resistance.[4-6] This has been described as one of the greatest challenges to public health today,[7,8] with the World Economic Forum placing antibiotic-resistant bacteria on the 2014 Global Risks List.[9] The five-year NIHR-funded 'TARGET' Programme for Applied Research was set up in 2010 to derive new knowledge to improve the management of antibiotics given to children presenting to primary care with RTIs and cough.

Systematic reviews from this programme [10,11] found that passive strategies targeting only parents, such as waiting room posters or pamphlets, do not appear to alter prescribing rates significantly. The most effective interventions involved targeting both parents and clinicians during a consultation, using automatic computer prompts for evidence-based prescribing, involving clinicians in the design of the intervention, using printed materials with actionable information and integrating interventions into routine clinical processes. Qualitative research found that communication within consultations often failed to meet parents' needs: information on symptom relief was lacking, safety-netting advice was too vague to be useful and parent concerns went unaddressed.[3,12-15] Clinicians revealed that prognostic uncertainty is an important driver of antibiotic prescribing, and would like information to help them identify the children at risk of future illness deterioration.[3,16,17]

Using a large multi-centre, prospective cohort study (over 8,300 children) we derived and internally validated a prognostic rule, using symptoms, signs, and demographic characteristics to predict hospitalisation in the following 30 days among children presenting to primary care with acute cough and RTI.[18,19] Findings from across the TARGET programme were synthesised to develop a complex intervention, designed to reduce antibiotic prescribing. The CHICO (Children's Cough) feasibility trial reported here is the final element of the TARGET programme, the aim of which was to assess the recruitment and acceptability of the complex intervention and to understand whether it is feasible to conduct a larger trial.

For peer review only - http://bmjopen.bmj.com/site/about/guidelines.xhtml

Methods

Design

The CHICO feasibility trial was a primary care cluster randomised controlled trial (RCT) comparing a web-based intervention with usual care for children presenting to general practices with RTI and acute cough. The trial was approved by the North West-Haydock Research Ethics Committee, UK (reference number: 14/NW/1034, Trial registration: ISRCTN23547970, UKCRN study ID: 16891). The trial protocol[20] was devised according to the SPIRIT guidelines[21] for randomised controlled trials, reporting of the findings followed CONSORT guidelines for clustered trials[22] and below is a brief summary of the methodology.

Practice and patient recruitment

General practices were invited to participate in the trial from locations in Bristol and the surrounding areas; the only exclusion criterion was for those few practices using very outdated internet browsers. General Practitioners (GPs) and prescribing nurses (hereafter termed ‘clinicians’) were eligible to recruit children to the trial and informed consent was obtained from the parent or legal guardian (carers). Children were included if they were aged between 3 months and under 12 years and presenting with a RTI with acute cough of no more than 28 days duration prior to consultation. They were eligible if they presented with illnesses such as asthma (including those with infective exacerbations), epilepsy or diabetes. Children who required same day hospital assessment or admission were also included. Children were excluded if they were considered to have a high risk of serious infection (immune-compromised, cystic fibrosis, splenectomy).

Randomisation and sample size

The allocation process was a one-off randomisation stratified for both practice size and prevalence of antibiotic prescribing using a proxy measure of amoxicillin suspension prescriptions (the main antibiotic used for children with cough) within the previous 12 months. As this was a feasibility trial, a formal (effectiveness-based) sample size calculation was not required, although we were interested in the recruitment rate to inform a larger trial design. Recruitment rates varied widely between practices in our previous cohort study[19] so we monitored recruitment levels closely and capped recruitment at no more than 30 patients per practice. Estimates from the cohort study suggested 15 practices in each arm of the trial would yield a pragmatic sample between 300 and 500 patients over a seven-month period.

Intervention

Intervention development will be described in more detail elsewhere, though the paper reporting algorithm development has been published.[19] In brief, findings from the TARGET programme were

For peer review only - http://bmjopen.bmj.com/site/about/guidelines.xhtml

synthesised using Green and Krueter's Precede-Proceed model [23] as a framework. The active elements of the intervention were determined to be elicitation of parent concerns, active no-antibiotic messages to the clinician, reduction of clinician uncertainty, and support for a no-antibiotic treatment response. These elements were provided using a within-consultation interactive web-based tool which also provided a data collection tool. This tool delivered the following intervention elements:

1. Recording children's symptoms and signs.
2. Elicitation and recording carers' concerns.
3. For each consultation children were identified as being at very low, normal or high risk of future hospitalisation; current NICE guidelines on antibiotic associated with each risk strata were also provided.
4. A personalised printout was produced for carers of each child. Clinical observations and carer concerns recorded in the system were used to produce this personalised information leaflet that explained the best home-care strategies and reinforced important safety netting advice. The aim of this leaflet was to give clinicians a tangible treatment action other than (or in addition to) prescribing, to improve safety-netting information and to provide home-care support for carers.

Data collection

Both control and intervention clinicians used the same study website to record carer consent, enter baseline and symptom data during the consultation and record their treatment decisions. Additionally intervention clinicians were provided with background information about how the risk algorithm was developed, recorded carer concerns, were given on-screen the future hospitalisation risk of the child and provided carers with a personalised printout. Use of the intervention was assessed by recording the number of times the clinicians used the web-based intervention and time spent on each page of the website. Follow-up data were collected each week from carers until the child's cough had resolved, or up to 8 weeks if not resolved. A medical record notes reviews were conducted at the recruiting practices to collect data relating to the 30 days following the recruitment consultation, including recruiting consultation timings, RTI-related antibiotic prescriptions, re-consultations, and RTI related hospitalisations.

Qualitative Interviews

Clinicians from both arms and carers from the intervention arm only were invited to participate in semi-structured interviews to explore their views of web-based data collection and the intervention. Interviews with carers were conducted either in the week following recruitment (to facilitate recall of the consultation) or after their child had recovered, to reflect on the whole follow-up data collection process. Purposive sampling was used to maximise variation in the sample of carers, including those with a range of child ages, home neighbourhood social-economic deprivation, illness severity scores,

For peer review only - http://bmjopen.bmj.com/site/about/guidelines.xhtml

and treatment outcomes. Clinicians were purposefully sampled to include variation in recruiting and prescribing levels.

Health Economics

The purpose of the economic analysis was to inform the feasibility and design of a within-trial economic evaluation alongside a larger RCT, including assessing web-based data collection as a means of gathering comprehensive resource use and quality of life data from carers. We adopted a health system (i.e. NHS) perspective for the analysis of the costs associated with resource use. We measured resources used from the time of recruitment until the earliest of either resolution or the end of the eighth week of follow-up. Health system resources used included GP consultations, use out-of-hours services, NHS 111, walk-in centres, and hospital and ambulance use. We examined quality of life using the Child Health Utility 9D (CHU-9D) instrument, a generic measure of paediatric quality of life validated for children aged 5-11 years. CHU-9D was included in the web-based tool for those children within the valid age-bracket.

Carers were given the option of providing data using a paper version rather than online if preferred. Participants were sent text and email reminders followed by telephone calls if the data were not returned promptly.[24,25] The length of the initial consultation was recorded electronically by the web-based tool and GP practice systems.

Data Analysis

Descriptive statistics were used to illustrate the characteristics of the carers, children and recruiting clinicians. For between-arm comparisons, categorical data were tested using Chi-square or Fisher's Exact test if an expected cell value was <5, continuous data were plotted and, if not normally distributed, non-parametric tests (Mann Whitney) were used. The use of baseline statistical tests to compare the arms is legitimate in the context of stratified cluster randomisation with subsequent (unconcealed) recruitment of individual participants. Such tests were used to ascertain if there was any evidence of differential recruitment of individuals across the trial arms beyond what would be expected by chance.

Qualitative interviews were audio-recorded, transcribed, anonymised and imported into NVIVO 10. Analysis of qualitative data began shortly after data collection started and was ongoing and iterative. An inductive thematic analysis approach was used to identify patterns and themes.[26] An initial coding frame was developed and refinded as new data were produced. Two researchers (CC and JH) double coded a subset of transcripts to inform the coding framework and ensure robust analysis.[27]

For peer review only - http://bmjopen.bmj.com/site/about/guidelines.xhtml

For both cost and quality of life data, the extent and nature of missing data was examined and compared between intervention and control practices. Descriptive statistics were used to explore resource use and cost by category and by arm to identify important cost drivers.

RESULTS

Ascertainment and data collection

A total of 32 practices were recruited to the trial, 16 randomised to each arm. Within the practices 104 clinicians signed up as recruiters and 64 recruited at least one child (18 nurses and 46 GPs). The target of 500 recruited children was achieved one month ahead of schedule (October 2014 to March 2015 inclusive); 542 children were invited to enter the trial and 501 agreed. Of the 41 not included, 18 (3.3%) declined, 19 (3.5%) did not meet the eligibility criteria and 2 (0.4%) were subsequently withdrawn by the carers. We obtained complete carer-reported follow-up data for 495 (98.8%) from the 501 children retained in the trial. Only two practices recruited no children; the median number of children recruited at each practices was 16 (range 0-30, IQR 4-29). Primary care medical record data were collected for 100% of the children. Web-based tool information was available for all but one of the 501 children.

Sample description

The median age of the 501 children in the trial was 2 years (IQR: 1-4 years, full range: 3 months-11 years), 49% were boys and there was no evidence of a difference in ethnicity when compared with 2011 UK Census data nor with the prevalence of smoking in the household when compared with a 2013 Opinions and Lifestyle survey. However, using the postcode of the family residence, the median IMD score in the trial (12.1) was markedly lower than national data (2010 Government statistics), suggesting children in this cohort were less deprived than children in the general population.

Comparison between study arms

Recruitment

Although there were fewer clinicians in the intervention arm (46 vs. 58 controls) they recruited 40% more children (292 vs. 209 children). Table 1 shows a lower proportion of control group clinicians recruited children (p=0.02). In the intervention arm a higher proportion of clinicians were nurses (28% vs. 19% controls), who seemed to recruit at a higher rate. More than half the children in the intervention arm were recruited by nurses compared with less than a third amongst the controls, although both GPs (median 3 children vs. 1 amongst controls, p=0.13) and nurses (median 12 children vs. 3 amongst controls, p=0.08) in the intervention arm recruited at higher rates compared with the controls.

For peer review only - http://bmjopen.bmj.com/site/about/guidelines.xhtml
For peer review only

Demographics

There was no marked difference between arms with regard to gender, ethnicity or deprivation score (Table 2). The median age of the children in the intervention group was younger compared to the control group (2 years vs. 3 years, p=0.03) and the intervention children lived in households with proportionally more smokers (29% vs. 17% controls, p=0.002).

Symptoms, signs and health outcomes

The majority of symptoms and signs were more severe amongst the intervention children (Table 3) who had a longer illness duration prior to consultation (p=0.03), a higher respiratory rate (p<0.001), higher wheeze prevalence (p=0.007), higher global illness severity scores, measured by both the carer (p=0.045) and clinician (p=0.02) and children where the clinician had a 'gut feeling' that something was wrong (p=0.03). There was little evidence of a difference between days to cough resolution (median 14 days, IQR 9-25 days in intervention arm compared with a median of 13 days, IQR 8-27days in the control arm, p=0.77) or follow-up appointments arranged during consultation (primary or secondary: 2.1% vs. 1.0% controls, p=0.33). There was a slightly higher proportion of children in the intervention arm attending Emergency Departments in the 30 days post recruitment (2.1% vs. 0.5% controls, p=0.14) although none of these children received antibiotic treatment. There was one hospital admission in the 30 days post recruitment; the child was in the intervention arm and was sent home the same day with a discharge diagnosis of 'viral induced wheeze'.

Antibiotic prescribing

As Table 4 shows, in the intervention group of the CHICO feasibility trial the overall antibiotic prescribing rate at consultation was 25% (19.9% immediate and 5.1% delayed) – demonstrating a marked reduction in both immediate and delayed prescribing compared with estimates from a few years earlier. However, the prescribing rate amongst the control children in the study was even lower (15.8%, 14.4% immediate and 1.4% delayed).

<u>Fidelity and acceptability of the intervention</u>

None of the data collection web-sessions during consultation was abandoned and no technical issues were reported with the website after resolving early teething problems associated with the use of older web-browsers. According to the available web page time-stamp data, the median time taken to complete the intervention was 5-6 minutes. This was consistent with clinicians reporting in the interviews that the intervention added 5-10 minutes onto their consultations. Some clinicians said they were reassured that hospitalisation risk agreed with their clinical judgement and some felt it was a useful backup for carers. The pages describing the background to the trial were only accessed on 29

For peer review only - http://bmjopen.bmj.com/site/about/guidelines.xhtml

occasions, although we are not able to discern whether these were unique visitors, or multiple visits from a few clinicians. Clinicians printed the personalised leaflet for the majority of the carers (92%) but only 4 of the 14 carers interviewed recalled receiving and reading the printout. No modifications were made to the intervention during the feasibility study.

Qualitative interviews with clinicians and carers

Interviews were conducted with 28 clinicians (17 general practitioners and 11 nurses) and 14 carers sampled with varying characteristics (Table 5). Selected quotes from the clinicians in each arm of the trial are listed in Table 6.

Recruitment

Carers perceived the data burden across the period of the study to be light. Clinicians, carers and children all liked the recruitment packs, which had been designed by a graphic designer, to be colourful and child-friendly. Clinicians in both arms reported recruiting less unwell children, for whom consultations were expected to be quicker to treat and therefore easier to combine with additional research activity (Table 6: Q1). Although clinicians commented that the intervention was easy and quick to use compared with other studies, it still added time to the consultation. Practices with an appointment system that channelled eligible children towards active recruiters, who were often minor illness nurses, had high recruitment rates. In contrast, practices that normally channelled same day appointments towards particular (non-recruiting) clinicians tended to be very low recruiters, despite attempts to get eligible patients redirected to recruiters (Table 6: Q2).

Using the intervention

Some clinicians in both arms initiated recruitment only after they had completed their ‘normal consultation’. Most said this was because engaging with the web-based tool interfered with their ‘normal consultation’, others that they needed to assess the child for serious illness before deciding whether to include them in the trial (Table 6: Q3). Clinicians reported a high degree of awareness of the problem of high antibiotic prescribing rates and described a range of other concurrent initiatives aimed at reducing antibiotic prescribing.

Intervention clinicians rarely reported using the CHICO algorithm results as a decision aid. Their reasons for this included: i) their ‘normal consultation’ including treatment decision was undertaken before initiating the intervention; ii) they decided early in the consultation that they didn’t need to prescribe and therefore extra information was not useful (Table 6: Q4) (perhaps exacerbated where they chose not to recruit sicker children); iii) they lacked trust in the algorithm (Table 6: Q5). Clinicians said the questions/information included in the intervention were more detailed than they usually asked, but none felt it had influenced or changed their decision. They were reassured when the

For peer review only - http://bmjopen.bmj.com/site/about/guidelines.xhtml

reported risk of hospitalisation agreed with their clinical judgement, and could be used as a useful backup to present to carers (Table 6: Q7). If the hospitalisation risk or advice provided was at odds with their clinical judgement a few clinicians said that they ignored the advice (Table 6: Q8).

Most carers said the CHICO consultation appeared as normal to them, while two said the history taking or explanations were more detailed. Few carers were aware of the reported risk of hospitalisation or subsequent advice from the CHICO intervention; one was reassured by the low risk advice. Two had experiences of the clinician prescribing antibiotics despite CHICO advice.

The carer leaflet

Clinicians valued having the leaflet to print out for carers highly; some said it was a useful alternative to a prescription. Some felt it backed up their recommendations and others felt it was good for carers to have printed information. Carers had mixed views about the leaflet; some felt it didn't tell them anything they didn't know already; others said it gave them some useful information; and in one case the carer reported that it prevented a re-consultation.

Health Economics

The review of patient practice records and the online data collection system, supported by telephone calls to carers, provided a comprehensive source of information on NHS resource use, although data missingness increased with symptom duration. Almost 99% of carers reported some NHS resource use. The initial consultation was estimated to take 15.29 minutes in the intervention arm, compared with 10 minutes in the control arm. Health system cost data were positively skewed, reflecting limited NHS costs other than for the initial GP consultation.

Mean NHS costs per patient from available cases (n=494) were £54.62 in control practices, and £78.78 in intervention practices. The difference of £24.15 is associated with a bias corrected and accelerated 95% confidence interval of £18.57 to £32.98. Mean per-patient costs were primarily determined by the costs of the initial consultation, which constitute 73% of NHS costs in the control arm and 78% in the intervention arm.

Only one in four recruited children were in the age range of 5-11 suitable for the CHU-9D instrument; moreover, only 44 (9% of all participants) provided baseline and follow-up data from the week of resolution to facilitate the estimation of quality adjusted life years (QALYs). The data on these children tentatively suggest that their quality of life returned to population norms after the baseline appointment, although this finding must be interpreted cautiously in light of the limited availability of this data.

For peer review only - http://bmjopen.bmj.com/site/about/guidelines.xhtml

For peer review only

Discussion

Summary of main results

The feasibility study showed high recruitment and retention. Quantitative and qualitative data confirm the intervention was acceptable to the clinicians and widely used, but often post-consultation and not within-consultation as intended. We found evidence of post-randomisation differential recruitment which is likely to have biased our estimates of intervention effect and explain our paradoxical results.

Evidence for differential recruitment included children in the intervention arm having more severe baseline characteristics than control children and higher recruitment rates in the intervention than control arm.

In the qualitative interviews, clinicians from both arms of the trial reported preferential recruitment of less unwell children as these were quicker to deal with and easier to combine with the research. This was particularly true in the control arm, where larger numbers of well children, relative to the intervention arm, were recruited. Evidencing Hawthorne effects (whereby clinicians modified their behaviour in response to their awareness of being in the trial) is challenging in RCTs without concurrent observational studies, but our 2011 to 2013 cohort study (in which we developed the algorithm)[19] showed antibiotic prescribing rates (37%) higher than both out intervention (25%) and control (16%) arms. It is therefore possible that Hawthorne effects may have been acting to reduce prescribing in both arms of our trial. Although we did not overtly advertise that the trial was focussing on reducing antibiotic prescribing, the clinicians may have inferred the aims of the trial. Our previous work with these practices may have alerted some clinicians to our aims, coupled with national and local campaigns to increase awareness of antimicrobial resistance. The control clinicians used the same web-based tool during consultation to record study data. Thus our data collection in itself amongst the controls may have inadvertently increased risk perception (by providing risk information, albeit partially obscured among the data collected that was previously unavailable to clinicians) as well as provide 'test' conditions.

The web-based data collection tool and review of medical notes were appropriate vehicles for resource-use data collection. The principal driver of higher NHS costs in the intervention group was longer initial GP appointments, when the web-based system was being used. There was little evidence on quality of life because of limited data availability.

Strengths and weaknesses

For peer review only - http://bmjopen.bmj.com/site/about/guidelines.xhtml

There are many strengths to this feasibility trial. Recruitment was so rapid that we stopped a month earlier than planned, with robust data collection and few missing values. We adequately resourced the trial to achieve excellent follow-up with little attrition, building up relationships with the families and using several types of communication media as an effective way of obtaining data for our web-based entry system. We reduced variation in practice recruitment levels, compared to our previous cohort study,[19] with a higher median number of patients per practice and fewer non-recruiting practices. None of the intervention web-sessions was abandoned and clinicians thought it was quick and easy to use compared to other interventions. Clinicians highly valued having the personalised leaflet as an alternative to a prescription.

Apart from differential recruitment and potential Hawthorne effects, we are aware of three other weaknesses. First, the intervention was designed to be used as part of a decision aid, but it became apparent that some clinicians only used the intervention after the treatment decision was made. The intervention might be more acceptable and easier to use if embedded within the existing practice records system. Second, using the intervention added around five minutes to consultation time, which could be reduced in future. Some of this time was to record research data and consideration needs to be given as to whether these data can be collected outside the consultation either from the medical notes or at practice level. Finally, although clinicians liked the personalised leaflets, carers were less enthusiastic. Further development through additional parent consultation would be recommended for a future trial.

Results in context with other research

We have constructed a complex intervention associated with lower antibiotic prescribing rates (from 37% to 25%) compared with data from similar practices in the same area a couple of years earlier[19], but an even lower rate among concurrent controls (16%). Local data over a four-year period suggest the extremely low prescribing rates among the control practices do not reflect usual practice, which may be due to differential recruitment.

Antibiotic prescription data from a large primary care database, covering 537 UK general practices during 1995–2011 suggest the rate of prescribing for respiratory tract infections in children and adults initially fell around the millennium but by 2011 had risen to 40%.[28] Our estimate of 37% amongst children from a large cohort study conducted between 2011 and 2013[19] is a robust estimate and a reduction in prescribing by almost a third to 25% is worth pursuing. To do this, a future trial must overcome the dual problems of differential recruitment and Hawthorne effects.

A primary care feasibility trial of patients with back pain[29] that also experienced differential recruitment recommended identifying patients, using independent researchers, before randomisation.

For peer review only - http://bmjopen.bmj.com/site/about/guidelines.xhtml

However, back pain is a chronic condition giving research teams time to identify and invite patient participation. The acute nature of childhood respiratory infections prevent prior identification and the cost of using independent researchers at multiple primary care sites would be prohibitive. One approach would be to select primary care sites that adopt a triage system for children with cough and work more closely with these different systems to help reduce selection bias. An alternative would be a ‘lighter touch’ design using practice level using routinely collected data and practice level consent, negating the need to consent and recruit individual patients post randomisation. The unit of interest would be the practice rather than the patient so all patients seen in a specified time would be included. Amoxicillin suspension is the main antibiotic given to children with acute cough and RTI and has been successfully used previously [30] as a proxy marker to assess antibiotic prescribing at the practice level.

The Hawthorne effect is not uncommon in clinical trials in general or indeed in trials specifically aimed at reducing antibiotic prescribing[31], but the mechanisms of effect and magnitude are not well understood.[32]

Conclusions

Many valuable lessons have been learnt from this feasibility study, and a redesign is required for any future trial. To negate differential recruitment and reduce the possibility of a Hawthorne effect a ‘light touch’ efficient design is needed that avoids patient recruitment at the clinician level and utilises data already routinely collected by the practices themselves. Better training in the use of the intervention and encouragement to use the tool as part of the consultation process would be facilitated if the intervention is embedded within current practice systems. Removing the need for clinicians to recruit patients or enter data other than that required for the tool would both reduce the time added to consultations and preserve usual practice amongst the control clinicians.

For peer review only

Competing interests

Financial competing interests. The authors declare that they have no competing interests. Non-financial competing interests. The authors declare that they have no competing interests.

Authors' contributions

ADH, PJL, PSB, NMR, JH, SH, NF, TJP and JI were responsible for developing the research question. PSB, ADH, JI, PJL, NMR, ST, SH, JH, CC, NF, AG, CJ and TJP were responsible for the study design and collection of data. ST, PSB and TJP were responsible for quantitative analysis, CC and JH were responsible for qualitative analysis, PD and SH were responsible for the economic evaluation. NMR and ST were responsible for study management, monitoring and co-ordination. PB and ST drafted the paper. All authors read, commented on and approved the final manuscript.

UK clinical research network (UKCRN) portfolio registration

The cohort study is registered on UK Clinical Research Network Portfolio as 'The CHICO (Children's Cough) Feasibility Trial' reference number 16891.

Funding statement

The TARGET Programme is funded by the National Institute for Health Research's Programme Grant for Applied Research Programme. This paper presents independent research funded by the National Institute for Health Research (NIHR) under its Programme Grant for Applied Research (Grant Reference Number RP-PG-0608-10018). The views expressed are those of the author(s) and not necessarily those of the NHS, the NIHR or the Department of Health.

NMR's time is currently supported by the National Institute for Health Research (NIHR) Collaboration for Leadership in Applied Health Research and Care West (CLAHRC West) at University Hospitals Bristol NHS Foundation Trust.

Data sharing statement

The full data set will be made available when all studies described within the protocol are complete and published. Application for the data to be released should be made in writing to Dr Peter Blair (PI for this feasibility study) via the Freedom of Information Officer at the University of Bristol

Acknowledgements

The authors are extremely grateful to the children, carers and families who have participated in the study, all GP practices including recruiting clinicians, administrative and research contacts and all other staff whose participation made this study possible. We would like to thank our parent and

For peer review only

clinician advisory groups for their time and thoughts (PAG members: Hannah Walter, Kelly McCauley, Tasha Buckland-Smith, Sandra Watt, Kathryn Johnson, Lucy Ashton, Louise Winter, Lee Williams, Liz Williams. GAG members: Elaine Luntz, Kirsty Alexander, Joanna Williams, Mike Rossdale, Knut Schroeder, Lucy Jenkins, Terry Kemple, Marion Steiner, Alex Hickson, Chris Milsom, Gina Wills, and Jo Jenkins). We thank colleagues from the TARGET Programme, the TARGET Programme Management Group and the TARGET Programme Steering Committee (Sandra Eldridge, Nick Francis, Paul Little, Joe Kai, Victoria Senior, Anna Thursby-Pelham and Jon Hayhurst) for their time, expertise and support. We are grateful to the following individuals who have helped with the study; Judy Cordell, Elizabeth Derodra, Sandra Mulligan, Sara Tonge and Hanna Oakes. Thanks goes to Kinetic Creative Limited for developing our web-based intervention in collaboration with the CHICO team. We are grateful for the support of the West of England Primary Care Network and the UK Clinical Research Networks involved in the study, specifically the West of England Clinical Research Network and the Business and Finance Officer Julie Brooks. The randomisation process for this trial was designed and delivered in collaboration with Daisy Gaunt and Chris Metcalfe from the Bristol Randomised Trials Collaboration (BRTC), a UKCRC Registered Clinical Trials Unit in receipt of National Institute for Health Research CTU support funding.

For peer review only

For peer review only - http://bmjopen.bmj.com/site/about/guidelines.xhtml

References

1) Hay AD, Heron J, Ness A, the ALSPAC study team. The prevalence of symptoms and consultations in pre-school children in the Avon Longitudinal Study of Parents and Children (ALSPAC): a prospective cohort study. *Fam Pract* 2005; 22(4):367-374.

2) Hollinghurst S, Gorst C, Fahey T, Hay A. Measuring the financial burden of acute cough in pre-school children: a cost of illness study. *BMC Family Practice* 2008; 9(1):10.

3) Lucas P, Cabral C, Hay A,el al. A systematic review of parent and clinician views and perceptions that influence prescribing decisions in relation to acute childhood infections in primary care. *Scandinavian Journal of Primary Health Care* 2015, **0**:1-10.

4) National Institute for Clinical Excellence (NICE): Department of Health. Respiratory tract infections: prescribing of antibiotics for self-limiting respiratory tract infections in adults and children in primary care. CG69. London: *National Institute for Clinical Excellence* 2008.

5) Department of Health. UK Five Year Antimicrobial Resistance Strategy 2013 to 2018. In: *Health* Do, ed., 2013.

6) Thompson PL, Spyridis N, Sharland M, et al. Changes in clinical indications for community antibiotic prescribing for children in the UK from 1996-2006: will the new NICE prescribing guidance on upper respiratory tract infections be ignored? *Arch Dis Child* 2009; **94**(5):337-340

7) World Health Organisation, Antimicrobial resistance: Global Report on Surveillance 2014, *World Health Organisation: France*. p. 256.

8) O'Neill, J., Tackling drug-resistant infections globally: final report and recommendations, in *Review on Antimicrobial Resistance*. 2016.

9) *World Economic Forum, Global Risks* 2014. 2014. p. 60

10) Vodicka TA, Thompson M, Lucas P, Heneghan C, Blair PS, Buckley DI, Redmond N, Hay AD; TARGET Programme team. Reducing antibiotic prescribing for children with respiratory tract infections in primary care: a systematic review. *Br J Gen Pract*. 2013 Jul; 63(612):e445-54.

11) Andrews T, Thompson M, Buckley DI, Heneghan C, Deyo R, Redmond N, Lucas PJ, Blair PS, Hay AD. Interventions to influence consulting and antibiotic use for acute respiratory tract infections in children: a systematic review and meta-analysis. *PLoS One.* 2012; 7(1):e30334.

12) Cabral, C. Ingram, J. Hay, A. and Horwood, J. “They just say everything's a virus” - parent's judgment of the credibility of clinician communication in primary care consultations for respiratory tract infections in children: A qualitative study. *Patient Education and Counseling* 2014; 95 (2), 248–253.

13) Ingram J, Cabral C, Hay AD, Lucas PJ, Horwood J. Parents' information needs, self-efficacy and influences on consulting for childhood respiratory tract infections: a qualitative study. *BMC Family Practice* 2013; 14:106

For peer review only - http://bmjopen.bmj.com/site/about/guidelines.xhtml

14) Cabral, CL, Horwood, JP, Hay, AD & Lucas, PJ. How communication affects prescription decisions in consultations for acute illness in children: a systematic review and meta-ethnography. *BMC Family Practice* 2014; 15:63.

15) Cabral, C. Ingram, JC, Lucas, PJ, Redmond, NM, Kai, J, Hay, AD & Horwood, JP. 'The influence of clinical communication on parents' antibiotic expectations for children with Respiratory Tract Infections'. *Annals of Family Medicine* 2016; 14(2): 141-147.

16) Cabral, C. Lucas, P.L. Ingram, J.C. Hay, A. Horwood, J. It's safer to... Safety for parents consulting and clinicians prescribing antibiotics for children with respiratory tract infections: an analysis across four linked qualitative studies. Social Science & Medicine 2015; 136-137:156- 164.

17) Horwood, J. Cabral, C. Hay, A. Ingram, J. Primary healthcare practitioners' diagnostic and antibiotic prescribing decisions in children's consultations for respiratory tract infections. British Journal of General practice Mar 2016; 66 (644): e207 -e213.

18) Redmond NM, Davies R, Christensen H, Blair PS et al (11). The TARGET cohort study protocol: a prospective primary care cohort study to derive and validate a clinical prediction rule to improve the targeting of antibiotics in children with respiratory tract illnesses. *BMC Health Serv Res*. 2013 Aug 17; 13(1):322.

19) Hay AD, Redmond NM, Turnbull S, Christensen H, Thornton, Little P, Thompson M, Delaney B, Lovering AM, Muir P, Leeming JP, Vipond B, Stuart B, Peters TJ, Blair PS. Development and internal validation of a clinical rule to improve antibiotic use in children presenting to primary with acute respiratory tract infection and cough: the 'TARGET' prognostic cohort study. *Lancet Respiratory Medicine* 2016 [in press].

20) Turnbull SL,, Redmond NM, Lucas P, Cabral C, Ingram J, Hollinghurst S, Hay AD, Peters, TJ, Horwood J, Little P, Francis N & Blair PS. The CHICO (Children's Cough) Trial protocol: a feasibility randomised controlled trial investigating the clinical and cost-effectiveness of a complex intervention to improve the management of children presenting to primary care with acute respiratory tract infection. *BMJ Open, 2015,* 5**,** e008615.

21) Chan AW, Tetzlaff JM,Gøtzsche PC, et al. SPIRIT 2013 explanation and elaboration: guidance for protocols of clinical trials.*BMJ* 2013; **346**: e7586

22) Campbell MK, Piaggio G, Elbourne DR, Altman DG; for the CONSORT Group. Consort 2010 statement: extension to cluster randomised trials. BMJ. 2012 Sep 4; 345:e5661. PMID: 22951546

23) Green LW, Kreuter M. Health Program Planning: An Educational and Ecological Approach. New York: McGraw-Hill; 2005.

24) Curtis L. Unit Costs of Health and Social Care 2013. Canterbury: Personal Social Services Research Unit; 2013.

25) Department of Health. Reference Costs 2012/13. London 2013.

26) Braun V, Clarke V. Using thematic analysis in psychology. *Qualitative Research in Psychology*. 2006; 3(2):77-101.

For peer review only - http://bmjopen.bmj.com/site/about/guidelines.xhtml

27) Meyrick J. What is good qualitative research? A first step towards a comprehensive approach to judging rigour/quality. *J Health Psychol*. 2006; 11(5):799-808

28) Hawker JI, Smith S, Smith GE, Morbey R, Johnson AP, Fleming DM, Shallcross L, Hayward AC. Trends in antibiotic prescribing in primary care for clinical syndromes subject to national recommendations to reduce antibiotic resistance, UK 1995-2011: analysis of a large database of primary care consultations. *J Antimicrob Chemother*. **2014** Dec; 69(12):3423-30.

29) Farrin A, Russell I, Torgerson D, Underwood M & Team UBT. Differential recruitment in a cluster randomized trial in primary care: the experience of the UK back pain, exercise, active management and manipulation (UK BEAM) feasibility study. *Clin Trials* 2005, 2, 119-24.

30) Butler CC, Simpson SA, Dunstan F et al. Effectiveness of multifaceted educational programme to reduce antibiotic dispensing in primary care: practice based randomised controlled trial. *BMJ* 2012, 344.

31) Mangion-Smith R, Elliott MN, McDonald L & McGlynn EA. An observational study of antibiotic prescribing behavior and the Hawthorne effect. Health Services Research 2002, 37, 1603-23.

32) McCambridge J, Witton J, Elbourne DR. Systematic review of the Hawthorne effect: new concepts are needed to study research participation effects. *J Clin Epidemiol* 2014, 67, 267-77.

review only

Table 1: Comparison of the clinician variables in the two arms of the trial

	Control		Intervention		
	n/N or Median	% or (IQR)	n/N or Median	% or (IQR)	p-value
**Clinician profile**					
Number of GCP trained clinicians	34/58	58.6%	32/46	69.6%	0.25[1]
Years since qualified	22	(14-27)	23	(17-27)	0.38[2]
**Clinicians who recruited**					
Number of recruiting clinicians	30/58	51.7%	34/46	73.9%	0.021[1]
Number of recruiting nurses	7/11	63.6%	11/13	84.6%	0.36[3]
Number of recruiting GPs	23/47	48.9%	23/33	69.7%	0.064[1]
**Number of children recruited by clinician type**					
Number of children recruited by nurses	65/209	31.1%	155/292	53.1%	<0.001[1]
Number of children recruited by GPs	144/209	68.9%	137/292	46.9%	<0.001[1]
**Recruitment of children**					
Median number of children recruited per clinician	1	(0-4)	3	(0-11)	0.014[2]
Median number recruited per nurse	3	(0-7)	12	(3-17)	0.13[2]
Median number recruited per GP	0	(0-4)	2	(0-4)	0.077[2]

1 Chi-square test
2 Mann Whitney test
3 Fisher's Exact test

Table 2: Demographic Profile in each arm of the trial

	Control		Intervention		p-value
	Median	IQR	Median	IQR	
Child age	3 years	1-5 years	2 years	1-4 years	0.028[1]
Home IMD* score	12.1	6.9-21.5	12.1	6.5-23.9	0.74[1]
	n/N	%	n/N	%	
Gender (male)	105/209	50.2%	139/292	47.6%	0.56[2]
Any smoker in household	34/206	16.5%	82/288	28.5%	0.020[2]
**Ethnicity:**					
White	187/209	89.5%	251/292	86.0%	
Mixed	8/209	3.8%	17/292	5.8%	0.72 (4d.f.)[2]
Asian or Asian British	7/209	3.4%	9/292	3.1%	
Black or Black British	5/209	2.4%	11/292	3.8%	
Other ethnic group	2/209	1.0%	4/292	1.4%	

* Index of multiple deprivation
1 Mann Whitney test
2 Chi-square test

For peer review only - http://bmjopen.bmj.com/site/about/guidelines.xhtml

Table 3: Comparison of children's symptoms and signs

	Control		Intervention		
	**Median**	**(IQR)**	**Median**	**(IQR)**	**p-value**
**Symptom or sign**					
Illness duration prior to consultation	5 days	(3-13)	7 days	(4-14)	0.034[1]
Clinician reported illness severity score (0-10)	3	(2-4)	3	(2-4)	0.022[1]
	**Mean**	**SD**	**Mean**	**SD**	
Carer reported illness severity score (0-10)	4.89	1.75	5.23	1.94	0.045[2]
	**n/N**	**%**	**n/N**	**%**	
Severe cough (24 hrs prior to consultation)	41/208	19.7%	79/292	27.1%	0.058
Severe fever (24 hrs prior to consultation)	15/208	7.2%	26/292	8.9%	0.50
Moderate or severe vomiting (24 hours prior)	18/208	8.7%	41/292	14.0%	0.066
High temperature (≥37.8°C)	29/208	13.9%	29/292	9.9%	0.17
High respiratory rate*	11/208	5.3%	44/292	15.1%	<0.001
Inter subcostal recession	5/208	2.4%	11/292	3.8%	0.39
Wheeze (auscultation)	17/208	8.2%	48/292	16.4%	0.007
Crackles or crepitations	37/208	17.8%	65/292	22.3%	0.22
Clinician had 'gut feeling' something was wrong	12/208	5.8%	34/292	11.6%	0.025

* Using age-related cut-offs
1 Mann Whitney test
2 T-Test
3 Chi-square test

Table 4: Antibiotics prescribed at consultation in the two arms of the trial

**Prescribing**	**Control**		**Intervention**		**p-value**
	N	%	N	%	
No antibiotics prescribed	176/209	84.3	219/292	75.0	0.018 (2 d.f.)
Immediate antibiotics prescribed	30/209	14.4	58/292	19.9	
Delayed antibiotics prescribed	3/209	1.4	15/292	5.1	

For peer review only

Table 5: Description of clinicians & carers sampled for the qualitative analysis

**Clinician sample**	**Characteristic**	N	%
Total clinicians in sample		28	100%
Study Arm	*Intervention*<br>*Control*	16<br>12	57.1%<br>42.9%
Clinician type	*GP*<br>*Nurse*	17<br>11	60.7%<br>39.3%
Clinician recruitment rate	*> 20 children*<br>*10-20 children*<br>*< 10 children*	5<br>11<br>12	17.9%<br>39.3%<br>42.9%
Clinician antibiotic Prescribing rate	>25%<br>15-24%<br><15%	9<br>9<br>10	32.1%<br>32.1%<br>35.7%
**Carer sample**	**Characteristic**	N	%
Total carers in sample		14	100%
IMD quintile*	*1 (most deprived)*<br>*2*<br>*3*<br>*4*<br>*5 (most affluent)*	1<br>5<br>2<br>3<br>3	7.1%<br>35.7%<br>14.3%<br>21.4%<br>21.4%
Child age (in years)	*<2*<br>*2-4*<br>*5+*	8<br>4<br>2	57.1%<br>28.6%<br>14.3%
Hospitalisation Risk and Treatment decision	*Low Risk – Home care*<br>*Low Risk – Delayed antibiotics*<br>*Low Risk – Immediate antibiotics*<br>*Medium Risk - Home care*<br>*Medium Risk - Delayed antibiotics*<br>*Medium Risk – Immediate antibiotics*	5<br>3<br>1<br>3<br>0<br>2	35.7%<br>21.4%<br>7.1%<br>21.4%<br>0.0%<br>14.3%

* Index of Multiple Deprivation based on the postcode of the family home

For peer review only

For peer review only - http://bmjopen.bmj.com/site/about/guidelines.xhtml

Table 6: Verbatim quotes from the clinicians

	Quotes (type of clinician, number, arm of trial)
Q1	"*if they were quite poorly I wouldn't be putting them into CHICO, because it would take me um… longer to do that consultation and examination and think about the plan of care*" (NP, #182, Intervention Arm)
Q2	"*in a busy duty surgery, whilst triaging, would just forget and would book them in as the normal route through the duty surgery or with a nurse practitioner. So there were definitely probably children being seen that were missed*." (GP, #207, Control Arm)
Q3	"*need to examine them first to make sure that they weren't acutely unwell*" (GP, #145, Intervention Arm)
Q4	"*generally by the time you've got to that [the odds ratios] you've given your advice, haven't you, and you've got a gut instinct that actually there isn't anything wrong with that child*." (NP, # 162, Intervention Arm)
Q5	"*I would say that the question that skewed the algorithm the most, in my opinion, was the vomiting one. I think a lot of times parents would be over-reporting severity of vomiting. And that's difficult, I know, because you're looking for subjective data from parents*." (GP, #133, Intervention Arm)
Q6	"*I don't think it influenced me in my antibiotic prescribing at all. … I'm very aware that we over-prescribe, but I think that, you know, like I said, I think there are times where I overrode your system regardless*." (NP, #104, Intervention Arm)
Q7	"*it was nice to have the reassurance that the algorithm backed me up, if I'd thought that they didn't need antibiotics…. And sometimes I used that to reassure parents as well. But… I knew what I was going to do based on history and examination already, as opposed to using the algorithm to dictate my choices of what I was going to do*." (NP, #133 Intervention Arm)
Q8	*"But there were some conflicting things where it said, "No antibiotics," and I was going to prescribe anyway. So I just said, "I know it says that, but actually I feel* [prescribing antibiotics] *is more appropriate," for whatever reasons.* (GP, #104, Intervention Arm)
NP is Nurse Practitioner, GP is General Practitioner	

For peer review only - http://bmjopen.bmj.com/site/about/guidelines.xhtml

COREQ Statement

Feasibility cluster randomised controlled trial of a within-consultation intervention to reduce antibiotic prescribing for children presenting to primary care with acute respiratory tract infection and cough

Domain 1: Research team and reflexivity

Personal Characteristics

1. Interviewer/facilitator. Which author/s conducted the interview or focus group?
Dr Christie Cabral

2. Credentials. What were the researcher's credentials? E.g. PhD, MD.
PhD

3. Occupation. What was their occupation at the time of the study?
Research Fellow

4. Gender. Was the researcher male or female?
Female.

5. Experience and training. What experience or training did the researcher have?
Extensive training in qualitative research methods and XX years' experience conducting qualitative research.

Relationship with participants

6. Relationship established. Was a relationship established prior to study commencement?
No

7. Participant knowledge of the interviewer. What did the participants know about the researcher? e.g. personal goals, reasons for doing the research?
The personal goals of the researcher were to complete the aims and objectives of the study only. The researcher had no personal goals or reasons for doing the research. As part of recruitment and gaining informed consent clinicians were fully informed about the aims and objectives of the study.

8. Interviewer characteristics. What characteristics were reported about the interviewer/facilitator? e.g. Bias, assumptions, reasons and interests in the research topic.
The interviewer (Christie Cabral) is a social scientist. Christie declared her academic standpoint to interviewees so that they were aware she was not from a medical background. We found this to be beneficial as Christie was able to ask 'naïve' questions and ask for clarification where needed – this produced some rich data and minimized bias.

Domain 2: study design

Theoretical framework

9. Methodological orientation and Theory. What methodological orientation was stated to underpin the study? e.g. grounded theory, discourse analysis, ethnography, phenomenology, content analysis
In the Data Analysis section we explain how we used semi-structured interviews and thematic analysis, this was informed by grounded theory and the constant comparison method.

Participant selection

For peer review only

10. Sampling. How were participants selected? e.g. purposive, convenience, consecutive, snowball.

Purposive sampling was used to select interviewees in order to attempt to capture maximum variation in views and experiences in order that they adequately reflect those of a range of carers and clinicians involved in the trial. For carers from the intervention arm, a purposive sample was drawn in relation to child age, home neighbourhood social-economic deprivation, illness severity scores, and treatment outcomes. For clinicians from both trial arms a purposive sample was drawn in relation to, study arm, recruitment rate, antibiotic prescribing rate and professional role.

11. Method of approach. How were participants approached? e.g. face-to-face, telephone, mail, email

Parents who had provided consent to be contacted for a qualitative interview were contacted by telephone at preferred time. Clinicians who had agreed to take part in a qualitative interview were contacted by email with a follow up phone call if they did not respond to email.

12. Sample size. How many participants were in the study?

We recruited 28 clinicians and 14 carers

13. Non-participation. How many people refused to participate or dropped out? Reasons

Of those invited to participate in an interview, 4 clinicians (2 from each arm) and 3 parents did not complete an interview. Clinicians gave being too busy as their reason for not participating. Parents simply did not respond to further attempts to contact them, so it was not possible to record their reason for no participating.

Setting

14. Setting of data collection. Where was the data collected? e.g. home, clinic, workplace

Interviews were conducted over the telephone.

15. Presence of non-participants. Was anyone else present besides the participants and researchers?

No

16. Description of sample. What are the important characteristics of the sample? e.g. demographic data, date.

Table 5 outlines a description of clinicians and carers sampled for the qualitative analysis. We interviewed 28 clinicians (17 GP's and 11 nurses) with 57% from the intervention arm. We interviewed 14 carers from the intervention arm selected from areas of high and low social-economic deprivation, who consulted for children with ranging in ages (8 children <2 years, 4 children between 2-4 years and 2 over 5 years) and had a range of treatment decisions.

Data collection

17. Interview guide. Were questions, prompts, guides provided by the authors? Was it pilot tested?

A semi-structured, topic guide used for all interviews. The focus of the topic guide was participants' views and experiences of the trial. The topic guide was used flexibly so that interviewees could raise other issues they felt were important and also allowed for in-depth exploration of emergent themes.

18. Repeat interviews.

We did not conduct repeat interviews

19. Audio/visual recording. Did the research use audio or visual recording to collect the data?

Yes audio recording was used throughout data collection.

For peer review only

20. Field notes. Were field notes made during and/or after the interview or focus group?
Brief field notes were made after the interviews.

21. Duration. What was the duration of the interviews or focus group?
Between 10 and 43 minutes

22. Data saturation. Was data saturation discussed?
Yes this was discussed in team meetings.

23. Transcripts returned. Were transcripts returned to participants for comment and/or correction?
No. We did not feel this was possible to offer in the time available in the study.

Domain 3: analysis and findings
Data analysis
24. Number of data coders. How many data coders coded the data?
Data was initially coded by the researcher Christie Cabral. Jeremy Horwood, a senior qualitative research fellow double coded a subset of 10% of the transcripts to inform the coding framework and ensure robust analysis. Interpretation of data was discussed regularly at multidisciplinary team meetings.

25. Description of the coding tree. Did authors provide a description of the coding tree?
No.

26. Derivation of themes. Were themes identified in advance or derived from the data?
Themes were derived inductively from the data.

27. Software. What software, if applicable, was used to manage the data?
We used NVivo 10 qualitative software package to manage the data.

28. Participant checking. Did participants provide feedback on the findings?
No.

29. Quotations presented. Were participant quotations presented to illustrate the themes / findings? Was each quotation identified? e.g. participant number
Yes, quotations to illustrate key themes are presented in Table 6.

30. Data and findings consistent. Was there consistency between the data presented and the findings?
Yes, we think so.

31. Clarity of major themes. Were major themes clearly presented in the findings?
Yes, we think so.

32. Clarity of minor themes. Is there a description of diverse cases or discussion of minor themes?
Yes, we think so.

For peer review only

Table 1: CONSORT 2010 checklist of information to include when reporting a cluster randomised trial

Section/Topic	Item No	Standard Checklist item	Extension for cluster designs	Page No *
**Title and abstract**				
	1a	Identification as a randomised trial in the title	Identification as a cluster randomised trial in the title	Yes (page 1)
	1b	Structured summary of trial design, methods, results, and conclusions (for specific guidance see CONSORT for abstracts)[1,2]	See table 2	Yes (page 2)
**Introduction**				
**Background and objectives**	2a	Scientific background and explanation of rationale	Rationale for using a cluster design	Yes (page 4). For this type of knowledge intervention trial clustering is self evident so explanation not provided (given the word limit)
	2b	Specific objectives or hypotheses	Whether objectives pertain to the the cluster level, the individual participant level or both	Yes (page 4) although this is a feasibility study so the main objectives surround issues of recruitment and acceptability of the intervention
**Methods**				
**Trial design**	3a	Description of trial design (such as parallel, factorial) including allocation ratio	Definition of cluster and description of how the design features apply to the clusters	Yes (page 5)
	3b	Important changes to methods after trial commencement (such as eligibility criteria), with reasons		No important changes made
**Participants**	4a	Eligibility criteria for	Eligibility criteria for clusters	Yes (page 5)

		participants		
	4b	Settings and locations where the data were collected		Yes (page 5)
**Interventions**	5	The interventions for each group with sufficient details to allow replication, including how and when they were actually administered	Whether interventions pertain to the cluster level, the individual participant level or both	Yes (page 5)
**Outcomes**	6a	Completely defined pre-specified primary and secondary outcome measures, including how and when they were assessed	Whether outcome measures pertain to the cluster level, the individual participant level or both	Yes (pages 6 and 7) although these refer specifically to the feasibility study
	6b	Any changes to trial outcomes after the trial commenced, with reasons		No changes made
**Sample size**	7a	How sample size was determined	Method of calculation, number of clusters(s) (and whether equal or unequal cluster sizes are assumed), cluster size, a coefficient of intracluster correlation (ICC or *k*), and an indication of its uncertainty	This was a feasibility trial so no formal sample size required although our pragmatic approach has been described (page 5)
	7b	When applicable, explanation of any interim analyses and stopping guidelines		Not applicable
**Randomisation:**				
**Sequence generation**	8a	Method used to generate the random allocation sequence		Yes (page 5)
	8b	Type of randomisation; details of any restriction (such as blocking and block size)	Details of stratification or matching if used	Yes (page 5)
**Allocation concealment mechanism**	9	Mechanism used to implement the random allocation sequence (such as sequentially numbered containers), describing any	Specification that allocation was based on clusters rather than individuals and whether allocation concealment (if any) was at the cluster level, the individual	Yes (page 5) Simple randomisation used at cluster level, a one-off

		steps taken to conceal the sequence until interventions were assigned	participant level or both	procedure
**Implementation**	10	Who generated the random allocation sequence, who enrolled participants, and who assigned participants to interventions	Replace by 10a, 10b and 10c	
	10a		Who generated the random allocation sequence, who enrolled clusters, and who assigned clusters to interventions	Yes (page 5)
	10b		Mechanism by which individual participants were included in clusters for the purposes of the trial (such as complete enumeration, random sampling)	Yes (page 5)
	10c		From whom consent was sought (representatives of the cluster, or individual cluster members, or both), and whether consent was sought before or after randomisation	Yes (page 5)
**Blinding**	11a	If done, who was blinded after assignment to interventions (for example, participants, care providers, those assessing outcomes) and how		Not applicable (blinding could not be used)
	11b	If relevant, description of the similarity of interventions		Not applicable
**Statistical methods**	12a	Statistical methods used to compare groups for primary and secondary outcomes	How clustering was taken into account	Yes (page 7)
	12b	Methods for additional analyses, such as subgroup analyses and adjusted analyses		Yes (page 7)
**Results**				

For peer review only

**Participant flow (a diagram is strongly recommended)**	13a	For each group, the numbers of participants who were randomly assigned, received intended treatment, and were analysed for the primary outcome	For each group, the numbers of clusters that were randomly assigned, received intended treatment, and were analysed for the primary outcome	Yes (page 8)
	13b	For each group, losses and exclusions after randomisation, together with reasons	For each group, losses and exclusions for both clusters and individual cluster members	Yes (page 8)
**Recruitment**	14a	Dates defining the periods of recruitment and follow-up		Yes (page 8)
	14b	Why the trial ended or was stopped		Not applicable
**Baseline data**	15	A table showing baseline demographic and clinical characteristics for each group	Baseline characteristics for the individual and cluster levels as applicable for each group	Yes (page 20)
**Numbers analysed**	16	For each group, number of participants (denominator) included in each analysis and whether the analysis was by original assigned groups	For each group, number of clusters included in each analysis	Yes (page 8 and page 20)
**Outcomes and estimation**	17a	For each primary and secondary outcome, results for each group, and the estimated effect size and its precision (such as 95% confidence interval)	Results at the individual or cluster level as applicable and a coefficient of intracluster correlation (ICC or k) for each primary outcome	Yes (page 21) but only to a point as this is a feasibility trial
	17b	For binary outcomes, presentation of both absolute and relative effect sizes is recommended		Yes (page 21)
**Ancillary analyses**	18	Results of any other analyses performed, including subgroup analyses and adjusted analyses, distinguishing pre-specified from exploratory		Not applicable, the main analysis surrounds recruitment issues and qualitative work as part of a feasibility study
**Harms**	19	All important harms or unintended effects in each		No adverse events to report

For peer review only

		group (for specific guidance see CONSORT for harms[3])		
**Discussion**				
**Limitations**	20	Trial limitations, addressing sources of potential bias, imprecision, and, if relevant, multiplicity of analyses		Yes (pages 12 and 13) and summarised as requested (page 3)
**Generalisability**	21	Generalisability (external validity, applicability) of the trial findings	Generalisability to clusters and/or individual participants (as relevant)	Yes (pages 13 and 14)
**Interpretation**	22	Interpretation consistent with results, balancing benefits and harms, and considering other relevant evidence		Yes (pages 13 and 14)
**Other information**				
**Registration**	23	Registration number and name of trial registry		Yes (page 15)
**Protocol**	24	Where the full trial protocol can be accessed, if available		The trial protocol paper is referenced (page 5)
**Funding**	25	Sources of funding and other support (such as supply of drugs), role of funders		Yes (page 15)

** Note: page numbers optional depending on journal requirements*

For peer review only

[1] Hopewell S, Clarke M, Moher D, Wager E, Middleton P, Altman DG, et al. CONSORT for reporting randomised trials in journal and conference abstracts. *Lancet* 2008, 371:281-283

[2] Hopewell S, Clarke M, Moher D, Wager E, Middleton P, Altman DG at al (2008) CONSORT for reporting randomized controlled trials in journal and conference abstracts: explanation and elaboration. *PLoS Med* 5(1): e20

[3] Ioannidis JP, Evans SJ, Gotzsche PC, O'Neill RT, Altman DG, Schulz K, Moher D. Better reporting of harms in randomized trials: an extension of the CONSORT statement. *Ann Intern Med* 2004; 141(10):781-788.

For peer review only
