# Supplementary material for: Feasibility cluster randomised controlled trial of a within-consultation intervention to reduce antibiotic prescribing for children presenting to primary care with acute respiratory tract infection and cough
Source: BMJ Open. 2017 May 9;7(5):e014506. doi: 10.1136/bmjopen-2016-014506 (PMC5623421; doi:10.1136/bmjopen-2016-014506)
Supplement: Reviewer comments [file bmjopen-2016-014506.reviewer_comments.pdf]

PEER REVIEW HISTORY

BMJ Open publishes all reviews undertaken for accepted manuscripts. Reviewers are asked to complete a checklist review form (**http://bmjopen.bmj.com/site/about/resources/checklist.pdf**) and are provided with free text boxes to elaborate on their assessment. These free text comments are reproduced below.

ARTICLE DETAILS

**TITLE (PROVISIONAL)**	Feasibility cluster randomised controlled trial of a within-consultation intervention to reduce antibiotic prescribing for children presenting to primary care with acute respiratory tract infection and cough
**AUTHORS**	Blair, Peter; Turnbull, Sophie; Ingram, Jenny; Redmond, Niamh; Lucas, Patricia; Cabral, Christie; Hollinghurst, Sandra; Dixon, Padraig; Peters, Tim; Horwood, Jeremy; Little, Paul; Francis, Nick A.; Gilbertson, Anna; Jameson, Catherine; Hay, Alastair

VERSION 1 - REVIEW

**REVIEWER**	Amanda McCullough and Chris Del Mar<br>Centre for Resrearch in Evidence Based Practice<br>Bond University<br>Gold Coast, Queensland 4229 Australia<br><br>We are both funded by the NHMRC for research into antimicrobial resistance in primary care
**REVIEW RETURNED**	28-Oct-2016

**GENERAL COMMENTS**	This is an astonishingly large feasibility study, 500 children with acute respiratory infection and cough conducted in the UK with a complex intervention aimed at the clinician (mostly GPs, but also a large proportion of nurses). Recruitment to the study was high, and attrition low.<br>The main result was interesting: antibiotic prescribing was paradoxically higher in the intervention group (25%) than control (16%). This is clearly from selection bias (children in the intervention group were sicker).<br>Lessons learned from this feasibility study were: 1) improvements in recruitment and allocation of patients are warranted before proceeding to the main trial; 2) the intervention itself requires alteration since the CHICO algorithm results were rarely used as intended.<br>There are no methodological concerns, and we keenly anticipate the findings of the forthcoming main trial.<br>However the reporting in this study should be more detailed:<br>1.<br>The intervention is described as complex, containing several elements. But it is difficult to gain an exact picture of what it was: the materials used; the algorithm itself; and how clinicians were prepared prior. Nor it is possible to find on-line (at least with the attempt we tried). Perhaps the original version could be resurrected for readers to examine in a link if interested. This is important if the main trial uses quite a different intervention, and what was reported here then becomes lost. Completion of a TIDieR checklist (http://www.equator-network.org/reporting-guidelines/tidier/) would help.

	Were any modifications made to the intervention during the feasibility study?<br><br>2. The qualitative research included in this paper is important in understanding some of the reasons for selection bias, and issues with intervention fidelity. But it is reported too briefly (for example, it would be difficult to replicate from this description: no copy of the topic guide, nor a summary of the main/minor themes, along with supporting quotes).<br><br>There seems to be a choice. Either remove these data from this manuscript and publish separately, or consider completing the COREQ checklist (http://www.equator-network.org/reporting-guidelines/coreq/) to highlight the additional information that should be included in this paper.<br><br>3. Similarly, the health economics data are also somewhat skimpily described: It was not completely clear whether the estimated costs were just those attributable to the NHS, or also included direct and indirect patient costs.<br><br>4. The discussion of results was also somewhat brief (despite the long write-up). Why did the controls reduce antibiotic prescribing by so much? Hawthorn effect is dismissed, but surely some speculation should be entertained, particularly as a new study is now in design.<br><br>5. The inclusion of the rate of antibiotic prescribing (37%) of the earlier cohort study in the results section is slightly misleading. It would be better to remove this and start this paragraph at “As Table 4 shows” (line 33, page 9).<br><br>Amanda McCullough and Chris Del Mar<br>Centre for Research in Evidence-Based Practice, Bond University, Australia

**REVIEWER**	Allison Cole<br>University of Washington<br>Seattle, WA, USA
**REVIEW RETURNED**	28-Nov-2016

**GENERAL COMMENTS**	Clearly written manuscript describing important findings from a RCT. The preliminary results suggest no improvement in rates of antibiotic prescribing in the intervention group and the authors identify several possible explanations for this. I agree that a more pragmatic clinical trial design, which enrolls and randomizes practices, eliminating possibility of differential patient recruitment and need for individual patient consent could potentially address these challenges.

VERSION 1 – AUTHOR RESPONSE

Reviewer 1.1:
The intervention is described as complex, containing several elements. But it is difficult to gain an exact picture of what it was: the materials used; the algorithm itself; and how clinicians were prepared prior. Nor it is possible to find on-line (at least with the attempt we tried). Perhaps the original version could be resurrected for readers to examine in a link if interested. This is important if the main trial uses quite a different intervention, and what was reported here then becomes lost. Completion of a

TIDieR checklist (http://www.equator-network.org/reporting-guidelines/tidier/) would help.
Were any modifications made to the intervention during the feasibility study?
Our Response:
As yet we do not want to provide a link to the intervention as we do not want to contaminate the control arm of a future study (currently being considered by the NIHR for funding). We also need evidence of whether it works and that no adverse effects are observed. A separate paper on the development of the intervention (which incorporates the TIDieR checklist) is in progress. The algorithm has previously been described and published (now referenced) and we have now provided some detail on the theory we used to create the intervention (p5 and p6). No modifications were made to the intervention during the feasibility study and this has now been stated (p10).

Reviewer 1.2:
The qualitative research included in this paper is important in understanding some of the reasons for selection bias, and issues with intervention fidelity. But it is reported too briefly (for example, it would be difficult to replicate from this description: no copy of the topic guide, nor a summary of the main/minor themes, along with supporting quotes).
There seems to be a choice. Either remove these data from this manuscript and publish separately, or consider completing the COREQ checklist (http://www.equator-network.org/reporting-guidelines/coreq/) to highlight the additional information that should be included in this paper.
Our Response:
We have now included an additional table with relevant quotes from the clinicians which is also alluded to in the text (P23). We have also provided the COREQ statement as a supplementary file (P10).

Reviewer 1.3:
Similarly, the health economics data are also somewhat skimpily described: It was not completely clear whether the estimated costs were just those attributable to the NHS, or also included direct and indirect patient costs.
Our Response:
We have now added more clarity to our health economics reporting including the attributes of the estimated cost (P7 and P11).

Reviewer 1.4:
The discussion of results was also somewhat brief (despite the long write-up). Why did the controls reduce antibiotic prescribing by so much? Hawthorn effect is dismissed, but surely some speculation should be entertained, particularly as a new study is now in design.
Our Response:
We have restructured the discussion section and have included more discussion of the Hawthorne effects (P12).

Reviewer 1.5:
The inclusion of the rate of antibiotic prescribing (37%) of the earlier cohort study in the results section is slightly misleading. It would be better to remove this and start this paragraph at “As Table 4 shows” (line 33, page 9).
Our Response:
Agreed and this has now been moved to the discussion (P12).

Reviewer 2.1:
Clearly written manuscript describing important findings from a RCT. The preliminary results suggest no improvement in rates of antibiotic prescribing in the intervention group and the authors identify several possible explanations for this. I agree that a more pragmatic clinical trial design, which enrolls and randomizes practices, eliminating possibility of differential patient recruitment and need for

individual patient consent could potentially address these challenges.
Our Response:
We thank Reviewer 2 and are happy that we seem to concur on the way forward.

VERSION 2 – REVIEW

**REVIEWER**	Chris Del Mar<br>Bond University<br>Australia<br><br>I am receipt of<br>1. an NHMRC grant examining antibiotic prescribing for acute respiratory infections in Australian primary care;<br>2. an NHMRC grant to support the editorial work of the Cochrane ARI Group.
**REVIEW RETURNED**	08-Feb-2017

**GENERAL COMMENTS**	The revisions are entirely satisfactory. This is an important paper.
